# Ventricular tachyarrhythmia treatment and prevention by subthreshold stimulation with stretchable epicardial multichannel electrode array

**DOI:** 10.1126/sciadv.adf6856

**Published:** 2023-03-31

**Authors:** Sung-Hyuk Sunwoo, Myung-Jin Cha, Sang Ihn Han, Hyejeong Kang, Ye Seul Cho, Da-Hae Yeom, Chan Soon Park, Na Kyeong Park, Seong Woo Choi, Sung Joon Kim, Gi Doo Cha, Dongjun Jung, Suji Choi, Seil Oh, Gi-Byoung Nam, Taeghwan Hyeon, Dae-Hyeong Kim, Seung-Pyo Lee

**Affiliations:** ^1^ Center for Nanoparticle Research, Institute for Basic Science (IBS), Seoul 08826, Republic of Korea.; ^2^ School of Chemical and Biological Engineering and Institute of Chemical Processes, Seoul National University, Seoul 08826, Republic of Korea.; ^3^ Institute of Radiation Medicine, Seoul National University Medical Research Center, Seoul 03080, Republic of Korea.; ^4^ Departments of Cardiology and Internal Medicine, Asan Medical Center, University of Ulsan College of Medicine, Seoul 05505, Republic of Korea.; ^5^ Division of Cardiology, Department of Internal Medicine, Seoul National University Hospital, Seoul 03080, Republic of Korea.; ^6^ Department of Physiology, Seoul National University College of Medicine, Seoul 03080, Republic of Korea.; ^7^ Department of Physiology, Dongguk University College of Medicine, Gyeongju 38066, Republic of Korea.; ^8^ Department of Internal Medicine, Seoul National University College of Medicine, Seoul 03080, Republic of Korea.; ^9^ Department of Materials Science and Engineering, Seoul National University, Seoul 08826, Republic of Korea.

## Abstract

The implantable cardioverter-defibrillator (ICD) is an effective method to prevent sudden cardiac death in high-risk patients. However, the transvenous lead is incompatible with large-area electrophysiological mapping and cannot accommodate selective multichannel precision stimulations. Moreover, it involves high-energy shocks, resulting in pain, myocardial damage, and recurrences of ventricular tachyarrhythmia (VTA). We present a method for VTA treatment based on subthreshold electrical stimulations using a stretchable epicardial multichannel electrode array, which does not disturb the normal contraction or electrical propagation of the ventricle. In rabbit models with myocardial infarction, the infarction was detected by mapping intracardiac electrograms with the stretchable epicardial multichannel electrode array. Then, VTAs could be terminated by sequential electrical stimuli from the epicardial multichannel electrode array beginning with low-energy subthreshold stimulations. Last, we used these subthreshold stimulations to prevent the occurrence of additional VTAs. The proposed protocol using the stretchable epicardial multichannel electrode array provides opportunities toward the development of innovative methods for painless ICD therapy.

## Teaser

Ventricular tachyarrhythmias can be managed by using epicardial multi-channel electrodes with subthreshold stimulation protocols.

## INTRODUCTION

Sudden cardiac death⁠, a major cause of mortality⁠, represents 20% of deaths in adults of westernized countries (*1*). Half of the sudden cardiac deaths are attributed to myocardial infarction (MI), followed by ventricular tachyarrhythmia (VTA) (*2*, *3*), and peri-infarct regions are well-known substrates of ventricular tachycardia (VT) or ventricular fibrillation (VF). Therefore, continuous local cardiac signal monitoring and immediate detection of VTA focus are critical for the treatment and prevention of sudden cardiac death, especially in high-risk patients with MI.

**Fig. 1.**
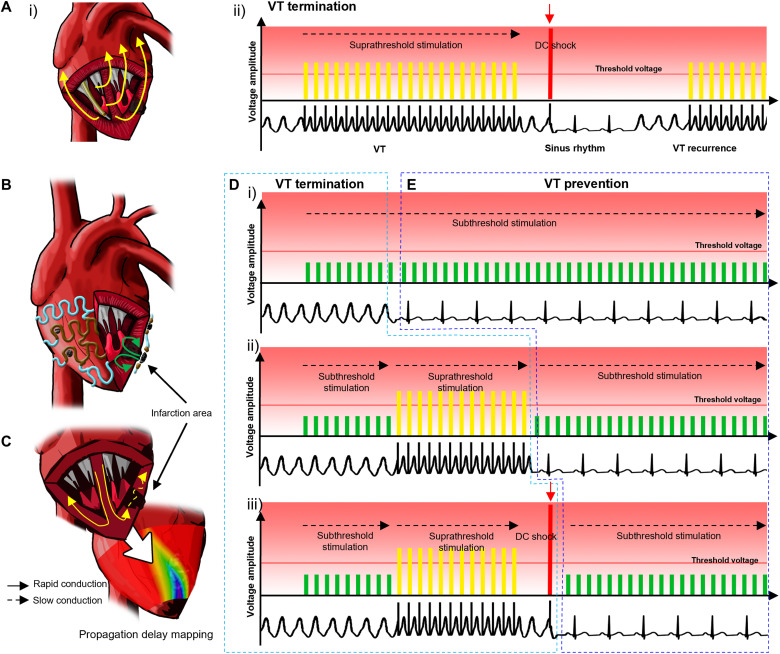
Stimulation and shock protocols for conventional ICD and stretchable epicardial multichannel electrode array. (**A**) Illustration of suprathreshold stimulation and/or shock protocol using a conventional ICD. (i) Suprathreshold stimulations are delivered via a single transvenous electrode. (ii) Sequential stimulation protocol for VTA termination used in current clinical practice based on suprathreshold stimulations (yellow) and DC shocks (red). VTA may recur, in which case the stimulation sequence is repeated until VTA termination. Surface electrocardiograms reveal the cardiac electrical activity during the abovementioned stimulations. (**B**) Illustration of a stretchable epicardial multichannel electrode array in a MI heart for mapping local epicardial electrograms and delivering stimulations. (**C**) Illustration demonstrating the epicardial mapping of the propagation delay in the infarction area. (**D**) Illustration explaining the sequential stimulations for VTA termination using the epicardial multichannel electrode array. (i) VTA termination after subthreshold stimulation (green). (ii) If subthreshold stimulation for VTA termination is unsuccessful, then suprathreshold stimulation (yellow) is applied to terminate the VTA. (iii) If both subthreshold and suprathreshold stimulations for VTA termination are unsuccessful, then the DC shock (red) is applied. (**E**) Illustration explaining constant subthreshold stimulations (green) for potential VTA prevention.

Implantable cardioverter-defibrillators (ICDs) can detect and terminate VTA by delivering suprathreshold stimulations (i.e., myocardial captured pacing) and/or high-energy electrical shocks through the transvenously implanted lead (Fig. 1A) (*4*). Despite the role of ICDs in preventing sudden cardiac death, there are large unmet clinical needs. The shock lead fixed on the right ventricular endocardium cannot accommodate multipoint or targeted pacing, and thus, nonspecific high-energy shocks propagating throughout the entire myocardium are unavoidable. The location of the lead is constrained by the cardiovascular structure (*5*). High-energy stimulation or shock significantly deteriorates patients’ quality of life (*6*), and repetitive shocks worsen cardiac function and patient prognosis (*7*). Text S1 describes the challenges of the current ICD-based treatment in more detail.

Subthreshold electrical stimulation, stimulation of the heart with a voltage lower than the capture threshold voltage needed for initiation of myocardial excitation, may be a potential solution for the VTA termination while avoiding disadvantages of high-energy stimulations and/or shocks (*8*–*11*). It does not interfere with normal myocardial contraction and, therefore, can terminate the VTA “quietly.” Suprathreshold stimulation, which is programmed as “antitachycardia pacing” on conventional ICDs, is advantageous over high-energy shock, as it has a lower incidence of side effects such as pain to the patient and electrical damages to the myocardium (*12*). The subthreshold stimulation can be advantageous further, even over the suprathreshold stimulation, as it is painless, does not interfere with normal myocardial contraction, and may not elicit any cardiac damages.

However, for successful subthreshold stimulation, it should be programmed and localized around the VTA focus because the subthreshold stimulation cannot propagate through the myocardium. Therefore, conventional ICDs with nontargeted lead position cannot accommodate the subthreshold stimulation. Meanwhile, the epicardial patch using intrinsically soft materials was recently reported to minimize mechanical stress on the dynamically beating heart (*13*), and the epicardial multichannel electrode arrays using stretchable conductive materials have been also reported (*14*–*16*). These mesh-type soft devices can envelop the entire ventricle to achieve multichannel recording and deliver programmed stimulations with precision. However, such soft epicardial electrodes have not been applied to the VTA management nor their efficacy tested and, thus, appropriately optimized stimulation protocols have hitherto not been developed.

Here, we used a stretchable epicardial electrode array (Fig. 1B) to provide local sequential stimulations as a treatment approach for VTA. First, we constructed an isochronal ventricular activation map using the epicardial electrode array to locate the substrate region of the VTA onset after MI (Fig. 1C) (*17*). Second, the sequential preprogrammed stimulations were delivered through the same epicardial electrodes to the selected region, especially the infarct region of the heart, for the VTA termination (Fig. 1D). Last, we showed the potential of the prophylactic subthreshold stimulation via the epicardial electrode array to suppress the onset of VTA (Fig. 1E). As a result, an innovative method based on subthreshold electrical stimulations could be developed for the detection, termination, and prevention of VTA using the stretchable epicardial multichannel electrode array.

## RESULTS

### Identification of the arrhythmogenic region using the stretchable epicardial multichannel electrode array

To treat fatal VTAs, it is crucial to understand how the normal electrical propagation is perturbed and where the arrhythmogenic focus is located. A device with multiple channels may enable the mapping of local cardiac electrical activities, which cannot be achieved by the conventional ICD used in clinical practice.

For the multichannel epicardial mapping of the intracardiac electrogram, a stretchable multichannel electrode array composed of silver nanowires and styrene-ethylene-butylene-styrene elastomer was fabricated and mounted on the epicardium of the subject animal (fig. S1, A to C). The signals recorded from the electrodes were transported to the data acquisition (DAQ) system through the stretchable interconnection and the anisotropic conductive film (fig. S1D). A composite of silver-gold core-shell nanowires, thermoplastic polyurethane (TPU), and poly(3,4-ethylenedioxythiophene):poly(styrene sulfonate) (PEDOT:PSS) can be also used to improve biocompatibility and reduce impedance of the electrode array (see text S2).

**Fig. 2.**
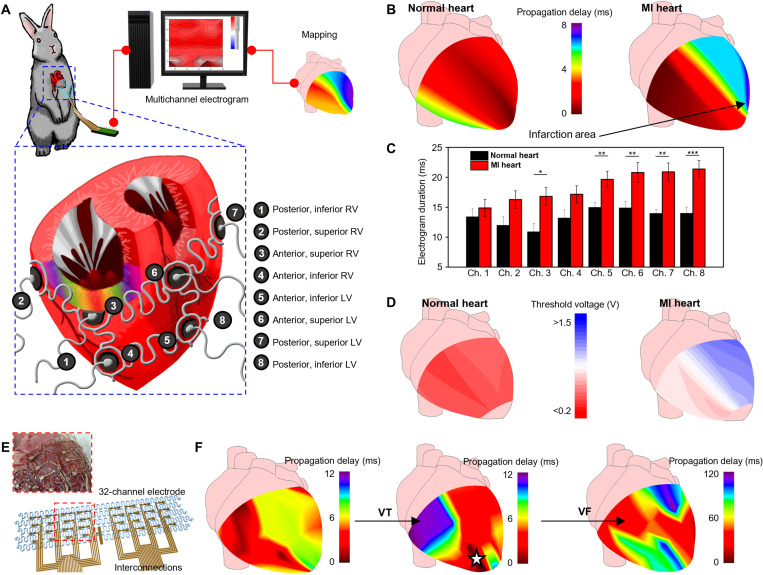
Mapping local epicardial electrograms from each location of the ventricle using the epicardial multichannel electrode array. **(A)** Illustrations of the epicardial multichannel electrode array wrapping the entire ventricle. We describe the eight ventricular locations for mapping. **(B)** Representative mapping image of the propagation time in the normal (left) and MI (right) hearts. **(C)** Local electrogram duration measured from the eight ventricular locations using the multichannel electrode array in the normal (black; *n* = 4) and MI (red; *n* = 8) hearts. *P < 0.05, **P < 0.01, and ***P < 0.001. **(D)** Representative mapping image of the local capture threshold voltages in the normal (left) and MI (right) hearts. **(E)** Schematic illustration of the 32-channel electrode. Inset image describes the 32-channel electrode mounted on the extracted heart. **(F)** Representative propagation mapping image of the MI heart under sinus rhythm (left), during VT (middle), and during VF (right) using the 32-channel electrode. During focal VT, arrhythmia spreads from the infarcted region (dark red region with white star), whereas chaotic propagation was observed during the VF in a fragmented manner.

These multichannel electrodes were placed on major anatomical locations of the ventricles and connected to the commercialized DAQ system to record the epicardial electrogram (Fig. 2A). The epicardial electrogram from each channel showed morphological differences between the normal rabbit hearts and the MI rabbit hearts. These differences on the epicardial electrograms were analyzed isochronally to identify the late activation zone in the ventricle, which corresponds to the infarct area, and most probably, the arrhythmogenic region (*18*). Sinus rhythms from the eight-channel electrodes exhibit activations of the corresponding regions in the ventricle at different time points. The time differences between the first activation in one region and the activations in other regions were calculated. This time difference was designated as the propagation delay, which was measured in the normal and MI rabbit hearts; the results were mapped as shown in Fig. 2B.

Mapping of the propagation delay in the normal heart showed that the epicardial electrical conduction starts from the interventricular area and spreads to the lateral regions of the ventricles (Fig. 2B, left). In contrast, the anterior region of the left ventricle (LV), typical location of MI after left anterior descending (LAD) coronary artery ligation, showed significant propagation delays by MI, thereby providing information on the location of the infarcted region based on the electrogram mapping (Fig. 2B, right). The prolonged local electrogram duration in the MI heart also showed that the electrophysiological properties of the entire ventricle change after MI (Fig. 2C) (*19*, *20*). The local electrogram duration was particularly prolonged in the LV, albeit prolonged in all channels. The prolonged local electrogram duration implies impaired ventricular conduction, supporting the reliability of the propagation mapping analysis using the epicardial multichannel electrode array.

In addition to the mapping of propagation delay and prolonged local electrogram duration, the capture threshold voltage of each ventricular region also supported the identification of the infarcted region (Fig. 2D). The capture threshold (*21*) was measured with burst pacing followed by monitoring of the subsequent electrogram and visual recognition of the heartbeat (fig. S2A). It is known that the capture threshold voltage tends to increase in the entire myocardium, more specifically in the infarcted region, of the ischemic heart (*22*). The normal heart demonstrated low capture threshold voltages across the entire ventricle [Fig. 2D (left) and fig. S2B]; however, there was an increase in the capture threshold voltage at the anterior region of LV in the MI heart [Fig. 2D (right) and fig. S2C]. Moreover, the overall capture threshold voltage of the MI heart was higher than that of the normal heart. The voltage levels used for subsequent subthreshold and suprathreshold stimulation experiments were set on the basis of the capture threshold voltage data. Because there were differences in the capture threshold voltages between individual animals, the strength-duration curves were measured in the respective normal and MI rabbit hearts to determine the appropriate strength of the stimulation (fig. S2, D and E).

Detecting the substrate of VT and mapping the ventricular activation pattern with electrophysiological study is the basis of VT treatment (*23*). Therefore, the local electrograms of VTs were recorded and analyzed using the eight-channel stretchable epicardial electrode array, thereby providing information on the VT propagation pattern. In the case of focal VT, the arrhythmia was initiated from a localized site in the ventricle (colored in dark red) and propagated to the neighboring region (fig. S3, left). On the contrary, in the case of reentrant VT, the arrhythmogenic myocardial activation propagated in a rotating manner (fig. S3, right).

This mapping strategy using the stretchable epicardial electrode array could be expanded to a 32-channel electrode array for a fine resolution mapping (Fig. 2E). The propagation map of the MI heart under normal sinus rhythm (left), VT (middle), and VF (right) were made using the 32-channel electrode array (Fig. 2F and fig. S4, A and B), by which the origin of the VT could be localized more accurately. The 32-channel mapping results could be also displayed as movies (movies S1 to S3). During the normal sinus rhythm (fig. S4C and movie S1), the rhythm was initiated from the top left side of the map [right ventricle (RV) base] and propagated to the bottom right side (LV apex). The LV area showed abnormally slowest conduction, which indicates a feature of the MI region. Under VT (fig. S4D and movie S2), however, the rhythm was initiated from the LV apex region, where the slowest conduction was observed under the sinus rhythm. The VT rhythm propagated to the RV apex, RV base, and then the LV base region in a rotational manner. Under VF (fig. S4E and movie S3), the rhythm was initiated from the LV apex region and propagated in a rotational manner as in the VT case, but the propagation rhythm was much more fragmented.

The current on-site arrhythmia identification is mostly limited to the use of single or biventricular lead devices, which does not enable fine mapping of the electrical signal of the heart during the arrhythmia. Currently, subsequent invasive electrophysiological studies are inevitable for direct mapping with a fine resolution. Here, our epicardial multichannel electrode array demonstrates the potential for performing a continuous, fine mapping of cardiac electrical activity using an implantable electrophysiological study device.

### Characterization of the subthreshold stimulation and suprathreshold pacing

**Fig. 3.**
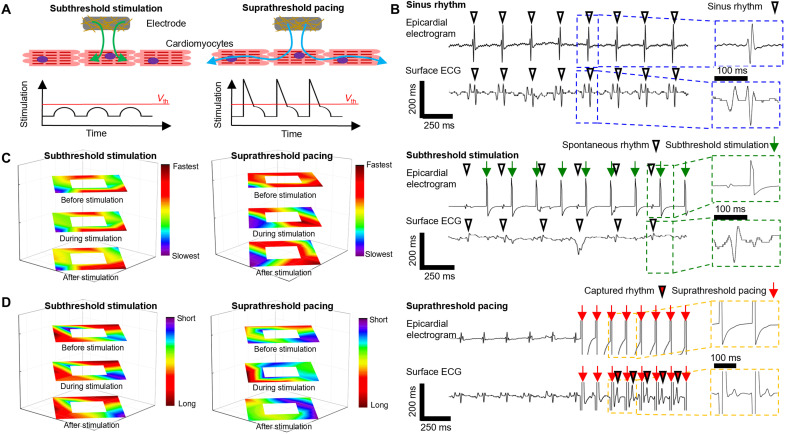
Comparison of the subthreshold stimulations and suprathreshold pacing. **(A)** Illustrations comparing the subthreshold stimulation and suprathreshold pacing. Subthreshold stimulation (left), electrical stimulation below the threshold voltage (*V*_th_), is nonpropagative stimulation that does not provoke myocardial activation in the neighborhood myocardium. Suprathreshold stimulation (right), electrical stimulation above the threshold voltage, is propagative stimulation that elicits subsequent myocardial activation in the neighborhood. **(B)** Representative epicardial electrogram and surface electrocardiogram (ECG) during the sinus rhythm (top), subthreshold stimulation (middle), and suprathreshold pacing (bottom). Under subthreshold stimulation (green arrow), spontaneous sinus rhythm was constantly observed (downward white triangle) in the surface ECG, while the captured rhythm (downward red triangle) was observed under suprathreshold pacing (red arrow). Although the stimulation was typically applied at a rate of 480 beats per minute (bpm), a stimulation rate of 600 bpm was also used if the cyclic length was shorter, and hence, the stimulation rates can be different (green arrows and red arrows). This was because the tendency of the VTA induction was different between each animal. A single dose of norepinephrine was enough for the VTA induction in some animals, while multiple doses were needed in other animals. **(C)** Propagation map of the rabbit heart, constructed with the 13-channel electrode array. The left image shows the subthreshold stimulation propagation map from the epicardium before stimulation (top), during stimulation (middle), and after stimulation (bottom). The right image shows the suprathreshold pacing propagation map from the epicardium before stimulation (top), during stimulation (middle), and after stimulation (bottom). **(D)** Electrogram duration map of the rabbit heart constructed with the 13-channel concentric electrode array under subthreshold stimulation (left) and suprathreshold pacing (right).

As previously mentioned in the capture threshold mapping (Fig. 2D and fig. S2), the voltage level of the subthreshold stimulation was determined as 50 to 75% of the threshold voltage and that of the suprathreshold pacing as 150 to 200%. These values were used throughout the entire study. The subthreshold stimulation did not induce myocardial excitation nor electrical propagation (Fig. 3A). To confirm that the subthreshold stimulation does not induce myocardial activation or electrical propagation, we fabricated a miniaturized electrode array with a single stimulation channel in the center and eight proximal and four distal recording channels arranged in a concentric shape (fig. S5A). The size of the stimulation electrode and the surrounding recording electrodes was 200 μm by 1000 μm and 200 μm by 300 μm, respectively. The distance between the neighboring electrodes was 600 μm. The characteristics of the subthreshold stimulation and the suprathreshold pacing were analyzed on the non-MI normal rabbit LV. The subthreshold stimulation (onset on green arrow) did not change the frequencies or morphologies of the spontaneous sinus beat (onset on empty arrowheads) of the nearby myocardium in the 12 surrounding regions where the recording channels are located (Fig. 3B, middle). However, the suprathreshold pacing (onset on red arrow) changed the electrogram of the entire electrode array significantly (Fig. 3B, lower panels). During the suprathreshold pacing, spontaneous sinus beats could not be observed.

The propagation map (Fig. 3C) and electrogram duration map (Fig. 3D) constructed with the data from the same, miniaturized electrode array (with one stimulating electrode in the center) during the subthreshold stimulation (left) and suprathreshold pacing (right) were also analyzed. The propagation pattern (Fig. 3C, left) and electrogram duration (Fig. 3D, left) did not change during and after the subthreshold stimulation, while the propagation pattern (Fig. 3C, right) and electrogram duration (Fig. 3D, right) changed markedly during and after the suprathreshold pacing. Detailed analyses comparing the propagation pattern and electrogram duration before, during, and after the subthreshold stimulation and suprathreshold pacing are presented in fig. S5 (B and C), as well as the ventricular activation by R-peak time in fig. S5D.

Note that all of these experiments demonstrating the effect of subthreshold stimulation were done in the normal heart, i.e., viable cells in the miniaturized electrode array with no infarct tissue. There may be a possibility of myocardial capture with exit block in these experiments, but this possibility would be insignificant, if any. On the basis of these results, we could verify that the subthreshold stimulation protocols used throughout the experiments do not elicit any electrical excitation or propagation in the myocardium of the highly localized stimulation site.

### Termination of VTA by electrical stimulations starting from subthreshold stimulation

**Fig. 4.**
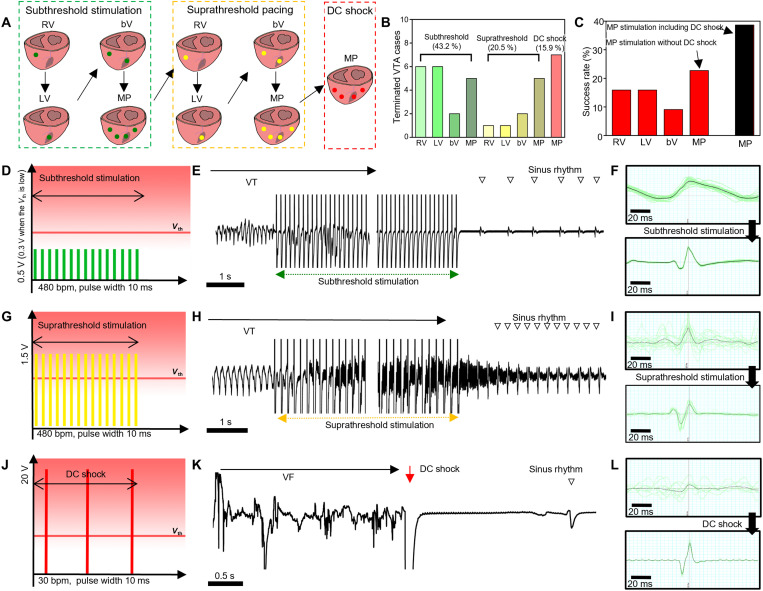
Stimulation protocol and in vivo results for VTA termination using the stretchable epicardial multichannel electrode array. **(A)** Illustrations describing the sequential stimulation protocol for VTA termination. Green, yellow, and red dots indicate the locations of subthreshold stimulation, suprathreshold pacing, and DC shock, respectively. **(B)** Success rate of VTA termination per stimulation step. **(C)** Success rate of VTA termination per stimulation location. **(D)** Schematic illustration of subthreshold stimulation. **(E)** Surface ECG acquired during subthreshold stimulation. Sinus rhythm (downward unfilled triangle) was restored after subthreshold stimulation. The middle part of the ECG tracing was abbreviated for a more concise presentation of the stimulation and the termination of VTA. **(F)** Normalized ECG peaks before (top) and after (bottom) subthreshold stimulation. The black line corresponds to the average morphology of 20 peaks (green lines). **(G)** Schematic illustration of suprathreshold stimulation. **(H)** Surface ECG acquired during suprathreshold stimulations. Normal, but faster, sinus rhythm (downward unfilled triangle) was restored after suprathreshold stimulation. The middle part of the ECG tracing was abbreviated for a more concise presentation of the stimulation and the termination of VTA. **(I)** Normalized ECG peaks before (top) and after (bottom) suprathreshold stimulation. The black line corresponds to the average morphology of 20 peaks (green lines). **(J)** Schematic illustration of DC shock. **(K)** Surface ECG acquired during DC shock. **(L)** Normalized ECG peaks before (top) and after (bottom) DC shock. The black line corresponds to the average morphology of 20 peaks (green lines). bV, biventricular; MP, multipolar.

We applied the subthreshold stimulation for the VTA termination by using the stretchable epicardial multichannel electrode array. This would alleviate the drawbacks associated with nonspecific high-voltage shocks in ICDs, whereas the multichannel array may accommodate precision stimulation with significantly less energy. A series of electrical stimulations, starting from local subthreshold stimulations, were applied to the target region of the ventricle to terminate VTA (Fig. 4A). The experimental protocol was designed to reduce the total energy delivered to the cardiac tissue and to minimize the potential side effects of high-energy electrical stimulations. Briefly, rapid subthreshold stimulations (Figs. 1D and 4A) were applied in the following sequence: subthreshold stimulation on the RV (non-MI region), subthreshold stimulation on the LV (MI region), biventricular subthreshold stimulation (two electrode pairs), and multipolar subthreshold stimulation (four electrode pairs). If the VTA was not terminated through the sequential subthreshold stimulations, then overdrive suprathreshold pacing (Figs. 1D and 4A) was applied in the abovementioned sequence. In the cases of unsuccessful VTA termination using suprathreshold pacing, multipolar DC shocks (Figs. 1D and 4A) were applied until VTA termination. Each sequence was applied for 10 s, and if unsuccessful, then the next sequence ensued. As the MI animal models were used to induce VTA throughout our study and it is well known that the majority of VTAs would be reentrant ischemic VTA initiating from the peri-infarct area, the stimulation sequence from RV to LV, followed by biventricular and multipolar pacing, was used to demonstrate that the currently used RV pacing in the clinical ICD would not be enough and that the stimulation in proximity to the infarction site (LV pacing) would be needed for subthreshold stimulation.

**Fig. 5.**
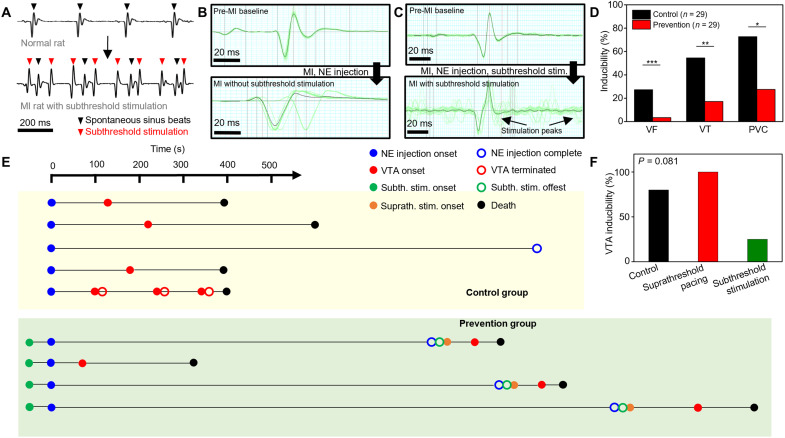
Prevention of potential VTAs using subthreshold stimulations. **(A)** Surface ECG from the prevention group rats before (top) and after (bottom) subthreshold stimulation. Black and red downward triangles indicate spontaneous sinus beats and subthreshold stimulation peaks, respectively. **(B)** Normalized rat surface ECG (black line) averaged from 20 peaks (green lines) in the control group before (top) and after (bottom) LAD coronary artery ligation and norepinephrine (NE) injection. **(C)** Normalized rat surface ECG (black line) averaged from 20 peaks (green lines) in the prevention group before (top) and after (bottom) LAD coronary artery ligation, norepinephrine injection, and subthreshold stimulation. **(D)** Inducibility of VTAs (VF, VT, and PVC) from the post-MI control group rats (black; *n* = 29) and post-MI prevention group rats (red; *n* = 29). *P < 0.05, **P < 0.01, and ***P < 0.001. **(E)** Timeline analysis of the VTA prevention experiments in rabbits. Starting from the norepinephrine injection, onset (filled circle) and offset (blank circle) time of the norepinephrine injection (blue), each VTA event (red), subthreshold stimulation (green), and suprathreshold pacing (orange) were marked on the timeline. The moment of death was marked as a black circle. **(F)** Graph comparing the VTA inducibility of the control group rabbit (black bar), subthreshold stimulation sequence of prevention group rabbit (green bar), and the suprathreshold pacing group rabbit (red bar).

Subthreshold stimulation does not impede ventricular contraction. Hence, it may not only terminate VTAs but also prevent potential VTAs in patients at high risk of fatal VTAs. We, therefore, evaluated whether the subthreshold stimulation could prevent VTA incidence. Post-MI rats (*n* = 58) were divided into either the control (*n* = 29) or prevention (*n* = 29) group. In the prevention group, the overdrive subthreshold stimulation was applied via the epicardial electrode array to the ventricle after MI induction by LAD coronary artery ligation (Fig. 5A). In contrast, the control group rats were not treated after the same ligation procedure. The inducibility of premature ventricular contraction (PVC) (*27*), VT, and VF was monitored in the two groups after a stepwise injection of high-dose norepinephrine (1 mg/kg per dose, up to 10 mg/kg).

There was a significantly higher chance of VTA occurrence in the post-MI control group after the norepinephrine injection (Fig. 5B), while VTA occurrence was smaller in the post-MI prevention group even after the serial injection of high-dose norepinephrine (Fig. 5C). Subthreshold stimulations did not impair the normal electrical activity propagation, and therefore, the subthreshold stimulation peaks could be observed between the sinus rhythms without any interruption of normal sinus beats (Fig. 5A).

The inducibility of spontaneous PVC, VT, and VF was significantly higher in the post-MI control group than in the post-MI prevention group (Fig. 5D). PVCs were developed in 28% (5 of 29 rats) and 76% (22 of 29 rats) of rats in the prevention and control groups, respectively. The inducibility of VT in the control group (16 of 29 rats; 55%) was three times higher than that in the prevention group (5 of 29 rats; 17%). Moreover, 1 (3%) and 8 (28%) of 29 rats in the prevention and control groups developed VF, respectively. Representative longitudinal surface electrocardiograms (ECGs) of both groups are shown in fig. S8 (A and B).

Two interesting observations were noted during the prevention experiments. First, four rats in the prevention group did not develop VTA during the subthreshold stimulation. However, VF occurred immediately after discontinuation of the subthreshold stimulation (fig. S8C). Hence, the subthreshold stimulation may suppress VTA initiation. Second, four rats in the control group developed recurrent VTAs. However, these VTAs were successfully terminated by the subthreshold stimulation (fig. S8D). Therefore, the unstable myocardial condition in the post-MI rats could be stabilized using subthreshold stimulation. More details regarding the incidence of events in the VTA prevention experiments are described in fig. S9.

To verify the potential of subthreshold stimulation to prevent the VTA onset in larger animals, similar experiments were performed on MI rabbits (Fig. 5E). For the post-MI control group (*n* = 5), excessive norepinephrine was injected intravenously (blue dot, filled) without electrical stimulations, as in the rat experiments. For the post-MI prevention group (*n* = 4), excessive norepinephrine was injected and the subthreshold stimulation (green dot, filled) was applied on the MI region of LV. In the control group, four of five rabbits (80%) showed VTA (red dot, filled) within 300 s of the norepinephrine injection, and three of four events (75%) were fatal (black dot; fig. S10A). One rabbit showed nonsustained VT, which was spontaneously terminated within 30 s (red dot, unfilled), but VT recurred afterward two more times before death. In the prevention group, one of four rabbits (25%) showed a fatal VTA event, while three rabbits showed no VTA event until the maximal dose of norepinephrine was injected (blue dot, unfilled). After terminating the subthreshold stimulation (green dot, unfilled) for the three surviving rabbits in the post-MI prevention group, suprathreshold pacing (orange dot) was applied to the same region of the MI heart (fig. S10B). The suprathreshold pacing evoked fatal VTA in 167.67 s (± 96.47) for all cases. The VTA inducibility of the suprathreshold pacing (three of three) was similar to that of the control group (four of five), while the VTA inducibility of the subthreshold stimulation (25%) was much lower (Fig. 5F).

Currently, subthreshold stimulation is not widely used in clinical practice. Nevertheless, a few studies have shown that implementation of the subthreshold stimulation may decrease the requirement for ICD interventions and the VT occurrence (*28*). Furthermore, the VTA recurrence rate even after successful ablations remains considerable, and repetitive large-energy shocks are associated with poor patient quality of life and outcome (*29*). Therefore, prophylactic subthreshold stimulation may be a promising therapeutic option for the prevention of VTA recurrence, especially for those at high risk of recurrent fatal VTAs.

## DISCUSSION

Using the stretchable epicardial multichannel electrode array, we provide an attractable option to treat and prevent VTA while overcoming the limitations of arrhythmia treatment options available in clinics. First, the VTA substrate region was localized by ventricular mapping using the epicardial multichannel electrode array. Second, the detected VTAs could be terminated by sequential stimulations using the electrode array, starting from the subthreshold stimulations that do not impede normal mechanical contractions and electrical propagations. Third, the constant subthreshold stimulation could potentially prevent VTA initiation. Collectively, these demonstrate the potential to use this stretchable epicardial multichannel electrode array to monitor, treat, and prevent VTA in those at a high risk of life-threatening arrhythmia.

Although this electrode array is versatile and can be refined to accommodate finer mapping, there are certain hurdles to overcome to test its utility in the future preclinical setting. First, as we demonstrated the potential benefit of subthreshold stimulation in the proximity of the VTA site, the subthreshold stimulation should be directly applied to stimulate the arrhythmogenic site from the beginning in future works. Second, a more detailed stimulation protocol that could find the most optimal stimulation duration, number of pacing pulses, and coupling intervals should be sought in the future. However, it should also be considered that the significance of the current work lies in the proof of concept by which it is possible to monitor and treat VTA without any perturbations in the natural myocardial excitations, rather than in the stimulation protocol itself. Third, we fixed the electrode array in its position with a simple suture that tied the device array around the heart. Although this fixation method was suitable for the current study that lasted less than 2 hours per animal after the application of the mesh, a mere simple suture may be far from best when trying to secure it on the epicardial surface for a long time. Specific fixing tools or methods such as a suspender device or an adhesive hydrogel might be needed for stable fixation of the mesh device for months or longer. The stability of the electrode array, i.e., change in the impedance and biocompatibility, needs further verification over the long-term period as well.

There are certain inherent limitations and challenges in the current clinical ICD devices. The current work demonstrates the potential utility of a epicardial multichannel electrode array combined with subthreshold stimulation protocols that would help improve the quality and efficacy of the antiarrhythmic treatment, with minimal detrimental effects to the heart and, ultimately, to the patient.

## MATERIALS AND METHODS

### Design of the stretchable epicardial multichannel electrode array

The eight-channel electrode array was designed to envelop the entire ventricular free walls. The electrode array includes recording/stimulating electrodes, ground electrodes, and interconnections (fig. S11). Among the eight recording/stimulating electrodes, four are placed on the RV, and the other four are placed on the LV. The ground electrodes cover both the LV and RV. Each recording/stimulating electrode has a circular shape with a diameter of 200 μm and is surrounded by the ground electrodes in a concentric fashion. The distance between the recording/stimulating and ground electrodes is 100 μm. The ground electrodes are linked with each other and with the external ground by interconnections. The serpentine-shaped design of the interconnection increases the stretchability of the array. Each recording/stimulating electrode is separately connected to a commercial DAQ system (Octal Bio Amp and PowerLab 16/35, AD Instruments, New Zealand). The fabrication process of the nanocomposite and the device is described in text S3.

### Animal preparation and coronary artery ligation to induce MI in rabbits

The mapping and treatment of VTAs were performed using New Zealand White male rabbits (average body weight, 2.5 kg). All animal experiments were approved by the Institutional Animal Care and Use Committee of Seoul National University Hospital [approval number: 18-0150-S1A3(1)]. Animals were maintained in a facility accredited by the Association for Assessment and Accreditation of Laboratory Animal Care (AAALAC) International in accordance with the *Guide for the Care and Use of Laboratory Animals* (eighth edition, NRC).

For induction of MI, LAD coronary artery ligation was performed 4 weeks before the antiarrhythmic stimulation experiments using the epicardial eight-channel electrode array. Rabbits were temporally anesthetized using an intramuscular injection of a 1:1 mixture of ketamine and Rompun (121.5 mg) and then intubated and ventilated with a mixture of room air and isoflurane for stable maintenance of anesthesia using a small animal ventilator (Harvard Apparatus 683, Harvard Bioscience Inc., MA, USA). Following a left thoracotomy between the third and fourth intercostal spaces, the proximal LAD coronary artery was ligated using a 6-0 black silk suture (Ailee Co. Ltd., Korea).

### Implantation of the epicardial electrode array and VTA induction in rabbits

Rabbits were anesthetized, and a horizontal incision was made between the abdominal and thoracic cavities, below the diaphragm. The diaphragm was dissected from the thorax to expose the thoracic cavity. After pericardiectomy, the epicardial eight-channel electrode array was overlaid on the heart to wrap the free walls of it. Both edges of the array were sutured with a 6-0 black silk suture. Intravenous access was established via the marginal ear vein for drug injection, while surface ECG signals were continuously monitored during the entire procedure.

The VTAs were induced in MI rabbits (4 weeks after ligation; *n* = 28) using continuous norepinephrine infusion via the marginal ear vein until significant surface ECG changes were noted. Moreover, chaotic overdrive electrical stimulations (frequency, amplitude, and pulse width of 10 Hz, 1.5 V, and 10 ms, respectively) were applied to the epicardium, via the epicardial electrode array, until a sustained VTA lasting for more than 30 s was detected.

### DCM models in rabbits

Doxorubicin was administered to New Zealand White male rabbits (average body weight, 2.5 kg; *n* = 2) via the marginal ear vein at a dose of 1 mg/kg twice per week to induce DCM. After 6 weeks of doxorubicin administration, we confirmed that the LV systolic function was significantly reduced by 20% of the baseline fractional shortening. The VTA induction and termination experiments were performed in these DCM rabbits as in an identical manner to that of the MI rabbits.

### Intracardiac electrogram mapping using the epicardial multichannel electrode array

Intracardiac electrograms and surface ECGs were recorded from the rabbits. After baseline local intracardiac electrogram recording, an overdrive pacing [240 beats per minute (bpm), 10 ms pulse width] was applied through the eight electrodes of the array to measure the capture thresholds from each stimulation site of the ventricle. The capture threshold was measured by reducing the stimulating voltage from and interval of 3.0 at 0.1 V. The capture state was determined by monitoring the subsequent electrogram and observing the absence of spontaneous beating during electrical stimulations. After VTA induction, we analyzed the VTA type, the earliest activation site during the event, and the propagation pattern. Stimulation treatments were initiated if the VTA lasted for more than 30 s. The overall mapping procedure was repeated in an identical manner with a multichannel electrode array of 32 channels where indicated to verify the potential of fine resolution mapping.

### Antiarrhythmic electrical stimulations for VTA termination

For subthreshold stimulation experiments to terminate VTAs, the stimulation rate was decided on the basis of the VTA cyclic length. Because the optimal duration, number, or coupling intervals of the anti-VTA pacing protocol are still uncertain in the current ICD system (*30*), the pacing protocol was simplified for the current study. Typically, stimulation was applied at a rate of 480 bpm; however, a stimulation rate of 600 bpm was used if the VTA rate was over 400 bpm. The subthreshold stimulation amplitude was decided on the basis of the capture threshold, which was typically 50 to 75% of the capture threshold voltage. The pulse width was 10 ms. Regarding the number and the site of the stimulation, a single-channel rapid stimulation was first applied to the non-MI area (RV) that would simulate a pacemaker used in clinical practice. If the VTA was not terminated with the RV stimulation, a single-channel overdrive stimulation was applied to the MI area (LV). If the VTA was not terminated, then a two-channel stimulation (one in the RV and LV each) was applied. If the VTA was not terminated, a four-channel stimulation (two channels in the RV and LV each) was applied. The sequential location in the pacing protocol (RV to LV sequence) was adopted because the reentrant VTA is known to be treated easily when a faster pacing is applied near the reentry circuit (*25*) and to show that the currently used RV pacing in the clinical ICD would not be compatible to accommodate subthreshold stimulation. The sequential method in the pacing protocol (subthreshold pacing to suprathreshold pacing and to DC shock) was adopted to minimize the amount of the shock and the potential damage to the heart and the patient.

If the VTA was not terminated with the abovementioned series of subthreshold stimulations, then suprathreshold pacing was applied in the same sequence and manner as that of the subthreshold stimulation. The amplitude of the suprathreshold pacing was generally 1.5 V, 150 to 200% of the capture threshold voltage, although 3 V was used if the capture threshold was higher than 1.5 V. The number and site of stimulation were identical in the sub- and suprathreshold stimulation protocols. Each subthreshold stimulation or suprathreshold pacing sequence was applied for 10 s, and if unsuccessful, the next sequence ensued.

If the VTA was not terminated with suprathreshold pacing, then a multichannel DC shock was applied through the four channels (two channels in the RV and LV each). A 20-V DC shock was applied at 30 bpm for 10 ms per shock and stopped when the sinus rhythm was restored. Successful VTA termination was defined as the end of VTA followed by at least three consecutive sinus rhythms.

### Animal preparation, device implantation, and experimental setup for rat experiments

All rat experiments were approved by the Institutional Animal Care and Use Committee of Seoul National University Hospital [approval number: 19-0116-S1A1(1)]. Animals were maintained in a facility accredited by AAALAC International in accordance with the *Guide for the Care and Use of Laboratory Animals* (eighth edition, NRC).

Eight-week-old Sprague-Dawley male rats (average body weight, 340 g) were anesthetized with the lowest possible dose of isoflurane inhalant (4% isoflurane mixed with oxygen), followed by endotracheal intubation and maintenance of 2 to 3% isoflurane (*31*, *32*). Each animal was connected to a small animal ventilator (RoVent Jr., Kent Scientific, USA). Full thoracotomy was performed as previously described for the rabbit model. After thoracotomy, the LAD coronary artery was ligated using a 7-0 black silk suture, accompanied by surface ECG recording using a conventional DAQ system, Quad Bio Amp and PowerLab 8/35 (AD Instruments, New Zealand).

### Subthreshold stimulation for potential VTA prevention in rats

After 4 weeks of inducing MI in rats, a single-channel epicardial electrode was mounted on the anterior surface of the rat heart. The rats were randomly divided into two groups: post-MI control (*n* = 29) and post-MI prevention (*n* = 29) groups. The overdrive subthreshold stimulation (rate, amplitude, and pulse width of 480 bpm, 0.1 V, and 10 ms, respectively) was applied only to the prevention group. To test the efficacy of prophylactic subthreshold stimulation on incident VTA, VTA was induced using high-dose norepinephrine (1 mg/ml) injection with concomitant subthreshold stimulation. Norepinephrine was continuously administered until VTA induction or until a cumulative norepinephrine injection dose of 10 mg/kg was attained (norepinephrine dose per animal weight).

### Prophylactic subthreshold stimulation to prevent VTAs on rabbits

After 4 weeks of inducing MI in rabbits with LAD coronary artery ligation, a single-channel epicardial electrode was mounted on the anterior surface of the rabbit heart LV, peri-infarct area, when applicable. The rabbits were randomly divided into two groups: post-MI control (*n* = 5) and post-MI prevention (*n* = 4) groups. The overdrive subthreshold stimulation (rate, amplitude, and pulse width of 480 bpm, 0.1 V, and 10 ms, respectively) was applied only to the prevention group. To test the efficacy of prophylactic subthreshold stimulation on incident VTA, VTA was induced using high-dose norepinephrine (1 mg/ml) injection with concomitant subthreshold stimulation. Norepinephrine was continuously administered until VTA induction or until a cumulative norepinephrine injection dose of 10 mg/kg was attained (norepinephrine dose per animal weight). No therapeutic trial was performed after the onset of VTA to show that the VTA was sustained and fatal. In addition, to compare the antiarrhythmic/proarrhythmic effect of the stimulation on the heart, overdrive suprathreshold pacing (rate, amplitude, and pulse width of 480 bpm, 2 V, and 10 ms, respectively) was delivered to the heart after termination of the subthreshold stimulation.

### Statistical analysis

Parameters acquired from the epicardial intracardiac electrogram, such as the electrogram duration, propagation difference, and R-peak time, were measured from the first eight consecutive beats with clear measurable signals. These were displayed as box plots with the mean and SDs and compared with either Mann-Whitney *U* test or Kruskal-Wallis test, as appropriate. The incidence of VTA inducibility was compared with chi-square test or Fisher’s exact test. Two-sided *P* values <0.05 were considered statistically significant and analyses were done with R version 4.0 (Vienna, Austria).

## Supplementary Materials

### This PDF file includes:

Supplementary Text Figs. S1 to S12 Legends for movies S1 to S3 References

## Notes

View/request a protocol for this paper from *Bio-protocol*.
