# Supplementary material for: Ventricular tachyarrhythmia treatment and prevention by subthreshold stimulation with stretchable epicardial multichannel electrode array
Source: Sci Adv. 2023 Mar 31;9(13):eadf6856. doi: 10.1126/sciadv.adf6856 (PMC10065438; doi:10.1126/sciadv.adf6856)
Supplement: Supplementary file 1 — Supplementary Text Figs. S1 to S12 Legends for movies S1 to S3 References [file sciadv.adf6856_sm.pdf]

Science Advances
AAAS

Supplementary Materials for

Ventricular tachyarrhythmia treatment and prevention by subthreshold stimulation with stretchable epicardial multichannel electrode array

Sung-Hyuk Sunwoo *et al.*

Corresponding author: Seung-Pyo Lee, sproll1@snu.ac.kr; Dae-Hyeong Kim, dkim98@snu.ac.kr; Taeghwan Hyeon, thyeon@snu.ac.kr

Sci. Adv. **9**, eadf6856 (2023)
DOI: 10.1126/sciadv.adf6856

The PDF file includes:

Supplementary Text
Figs. S1 to S12
Legends for movies S1 to S3
References

Other Supplementary Material for this manuscript includes the following:

Movies S1 to S3

Supplementary Texts

Text S1. Challenges of current antiarrhythmic devices

The use of implantable cardioverter-defibrillators (ICDs) has contributed to ventricular tachyarrhythmia (VTA) *(4)* management. As briefly described in the main text, conventional antiarrhythmic implantable devices have several drawbacks *(5, 33)*. Conventional ICDs use lead wire inserted transvenously into the right ventricle *(34, 35)*. Therefore, the location of the lead is constrained by the heart and the great vessel structures. Even in cardiac resynchronization therapies, lead locations are constrained by the cardiac and venous structure. Direct lead implantation into the left atrium or left ventricle (LV) via the aorta is not recommended because of the high blood pressure, high thromboembolic risk, and repetitive contraction of the chambers *(36-38)*. Therefore, direct local electrical stimulation to the LV is challenging because of the limitations in the pathways for lead wire insertion, albeit most myocardial scars are located in the LV.

Therefore, in order to electrically stimulate the infarcted LV region, high-energy electrical stimulations that non-selectively propagate across the entire ventricle are inevitable. However, this propagative high-energy current causes pain, complications *(6, 39)*, and myocardial damages that significantly affect patient satisfaction *(7, 39, 40)*. The stimulation-induced myocardial damages and the ensuing recurrent VTA events are not rare. Another notable drawback of the conventional transvenously implanted intracardiac devices is the mechanical stiffness of the lead, which potentially damages the dynamically beating myocardium *(41-43)*. It is reported that myocardial perforation due to the cardiac pacemaker leads, either microscopically or symptomatically, is not rare *(44-46)*. Moreover, a rigid lead in a vessel, valve, or heart chamber can interfere with the natural blood flow, leading to thrombosis or structural damage.

Text S2. Improvement of materials for the epicardial mesh electrode array

Materials with improved properties can be used to reduce impedance and improve electrode array biocompatibility. First, Ag-Au core-shell nanowires (Ag-Au NWs) and thermoplastic polyurethane (TPU) can be used instead of Ag NWs and SEBS, to improve biocompatibility *(15)*. The gold shell inhibits leaching of potentially toxic silver ions from the NWs. Although TPU is slightly stiffer than the SEBS elastomer, it has excellent biocompatibility *(47, 48)*. Second, a conductive polymer, poly(3,4-ethylenedioxythiophene):poly(styrenesulfonate) (PEDOT:PSS) can be added to the nanocomposite of Ag-Au NWs/TPU to reduce impedance *(49-51)*. The Ag-Au NW/PEDOT:PSS/TPU nanocomposite can be used to fabricate recording/stimulation electrodes, whereas the Ag-Au NWs/SEBS nanocomposite can be used to fabricate interconnections and ground electrodes (figs. S12A and S12B). The addition of PEDOT:PSS to the electrode improves electrochemical properties without significantly breaching the electrical conductivity (figs. S12C-F). Specifically, the nanocomposite with PEDOT:PSS shows a higher charge storage capacity of 32.8 mC/cm^2, while that of the nanocomposite without PEDOT:PSS is 10.1 mC/cm^2 (fig. S12F). Also, the impedance of the nanocomposite with PEDOT:PSS is ten-times lower than that of the nanocomposite without PEDOT:PSS (fig. S12G). The time-dependent change of the impedance, signal-to-noise ratio, and threshold voltage was also observed (figs. S12H-M). Although there was some performance decay, the overall material properties were maintained in a reasonable range for the 12 days of implantation. This modified electrode array showed successful *in vivo* recording and stimulation (figs. S12N and S12O).

Text S3. Supplementary methods

Fabrication of the electrode array using a composite of Ag NWs and SEBS

An elastomeric composite using Ag NWs and SEBS was used to fabricate the stretchable electrode array. The synthetic procedures of the Ag NWs are described in the previous reports *(15, 16)*. For the Ag NWs/SEBS composite, Ag NWs were dispersed in a solution of ethanol and toluene (volume ratio 1:2.5). The medical-grade SEBS elastomer, Tuftec^{TM} (Asahi Kasei, Japan), was dissolved in toluene (10 wt.%), and mixed with the Ag NWs solution.

The prepared composite solution was blade-coated on a poly-dimethylsulfoxane mold with a specific pattern, and air-dried on a 50°C hotplate. The Ag NWs/SEBS composite was used for the fabrication of the recording/stimulating electrode, interconnection, and ground electrode. The blade-coating procedure was repeated more than four times to obtain a sufficient thickness (350–500 μm).

The fabricated electrodes were detached from the molds for assembly (fig. S11). First, the recording/stimulating and ground electrodes were aligned on a SEBS substrate layer and affixed by applying heat (~135°C) and pressure (~12000N/m^2). The stretchable interconnections were aligned on the opposite side of the electrode array and affixed through the same procedure. Finally, the stretchable interconnections were connected to a customized printed circuit board (DITech, Korea) via an anisotropic conductive film (HST-9805-210, Elform Inc., USA). All channels were fabricated to have a similar electrical resistance of ~24 Ω (between the recording/stimulating electrodes and the printed circuit board pads).

Fabrication of the electrode array using a composite with improved biocompatibility and impedance

To enhance biocompatibility and to reduce contact impedance, materials other than Ag NWs and SEBS were used or added. For example, we used a composite of Ag-Au NWs, TPU, and PEDOT:PSS. Biocompatibility can be better improved by Ag-Au NWs and TPU *(52)* than by Ag NWs and SEBS. In addition, PEDOT:PSS was added to reduce the impedance of the composite *(48, 49)*.

To prepare the Ag-Au NWs/TPU/PEDOT:PSS composite, the re-dispersible PEDOT:PSS pellets (Merck, Germany) were dispersed in dimethylformamide (Samchun Chemicals, Korea) and mixed

with TPU (Tecoflex^{TM}, Lubrizol Co., USA) and Ag-Au NWs. The synthetic procedures of Ag-Au NWs were described in the previous reports *(15, 16)*. The mixture was vigorously stirred to obtain a homogeneous dispersion. After filling the solution in the mold and evaporating the solvents, the patterned composites were obtained. The method of device fabrication is identical to that used for the Ag NWs/SEBS composite.

Characterization of material properties of the composites

The electrical and electrochemical properties of the composite films (e.g., Ag-Au NWs/SEBS and Ag-Au NWs/TPU/PEDOT:PSS composites) were characterized as follows. The resistance of the Ag-Au NWs nanocomposite, Ag-Au NWs nanocomposite with PEDOT:PSS, and PEDOT:PSS films were measured using a sourcemeter (Keithley, USA). To evaluate long-term performance changes of the electrode, the electrode was subcutaneously implanted on the rat and extracted 4, 8, and 12 days after implantation (fig. S12). The performance of the extracted electrodes was compared with the non-implanted electrode. The impedance and current-voltage curve of the films (sliced in an equal shape of 5×5 mm, and fabricated with a thickness of 50 μm) were measured in a saline solution using an electrochemical analyzer (CHI660E, CH Instruments, Inc., USA).

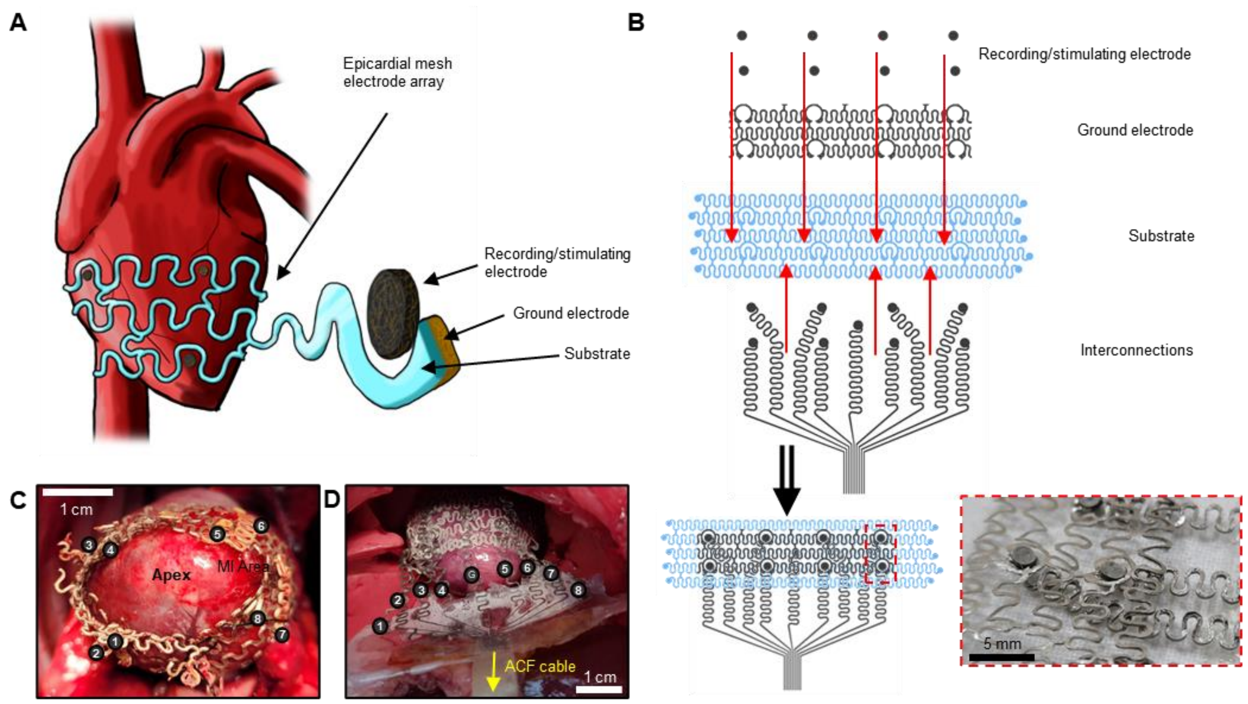

Fig. S1. Design of the stretchable epicardial multi-channel mesh electrode array and its implantation on the rabbit heart. (A) Stretchable epicardial multi-channel electrode array wrapping the ventricle. **(B)** Electrode array design and structure. The design of the recording/stimulating electrode, ground electrode, substrate, and interconnections are shown from top to bottom. Red arrows indicate the integration sequence of each layer. The red dotted box at the bottom right shows an optical image of the assembled electrode array. **(C)** Optical camera image showing an apical view of the electrode array wrapping the rabbit heart. Interconnections are removed to show electrode locations on the heart. The MI area is generally located near the channel 5, 6, and 8. **(D)** Optical camera image showing an anterior view of the electrode array wrapping the rabbit heart. The interconnection array is connected to the external data acquisition via an ACF.

(MI, myocardial infarction; ACF, anisotropic conductive film)

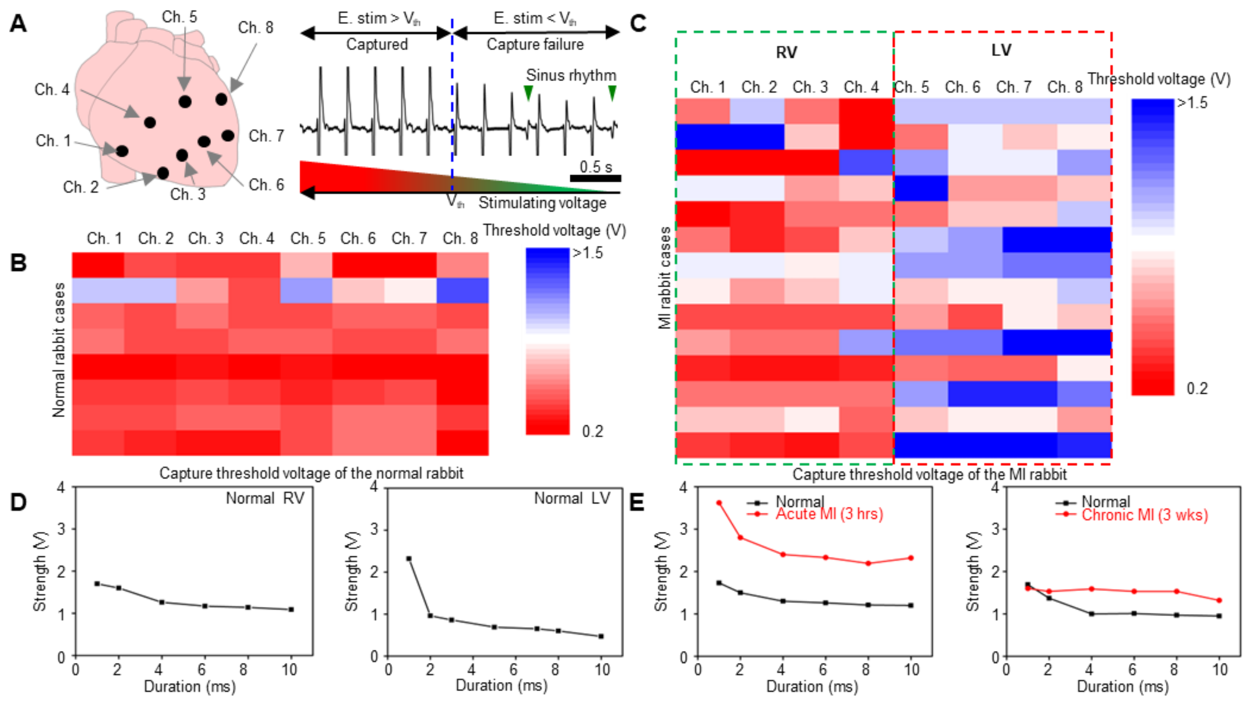

Fig. S2. Determination of the capture threshold voltages in the rabbit myocardial infarction models. (A) Schematic illustration explaining the capture threshold voltage on the rabbit heart. While electric stimulation was delivered via each electrode channel, the surface ECG was monitored to analyze whether the myocardial activation was captured or not. The stimulating voltage was reduced gradually until the spontaneous sinus rhythm (green downward triangle) was monitored. **(B)** Threshold voltage plots of the 8 normal rabbits. Channels 1-4 are located on the RV, while channels 5-8 on the LV. **(C)** Threshold voltage plots of the 14 MI rabbits. **(D)** Strength-duration curve of the normal rabbit heart RV (left) and LV (right). **(E)** Strength-duration curve of the normal rabbit (black) and MI rabbit (red). Left plot compares normal rabbit and 3 hours after its MI (acute MI). Right plot compares normal rabbit and 3 weeks after its MI (chronic MI).

(E.stim, electric stimulation; ECG, electrocardiogram; LV, left ventricle; MI, myocardial infarction; RV, right ventricle; V_{th}, threshold voltage)

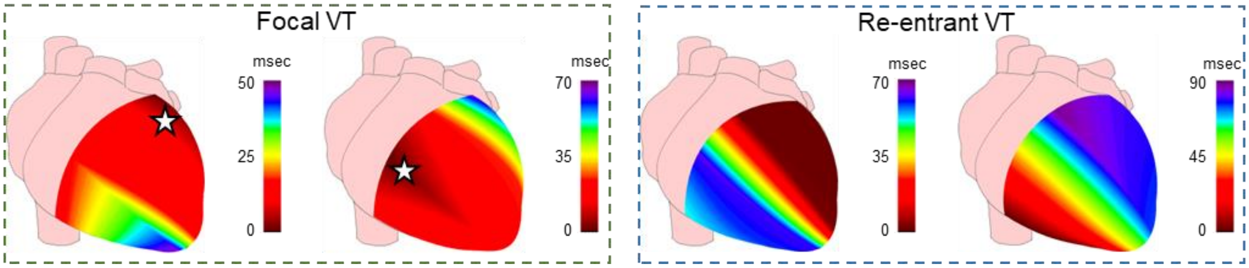

Fig. S3. Propagation map of the various ventricular tachycardias. The propagation delays are projected on the cardiac map. White stars indicate potential substrate areas of focal VT.
(VT, ventricular tachycardia)

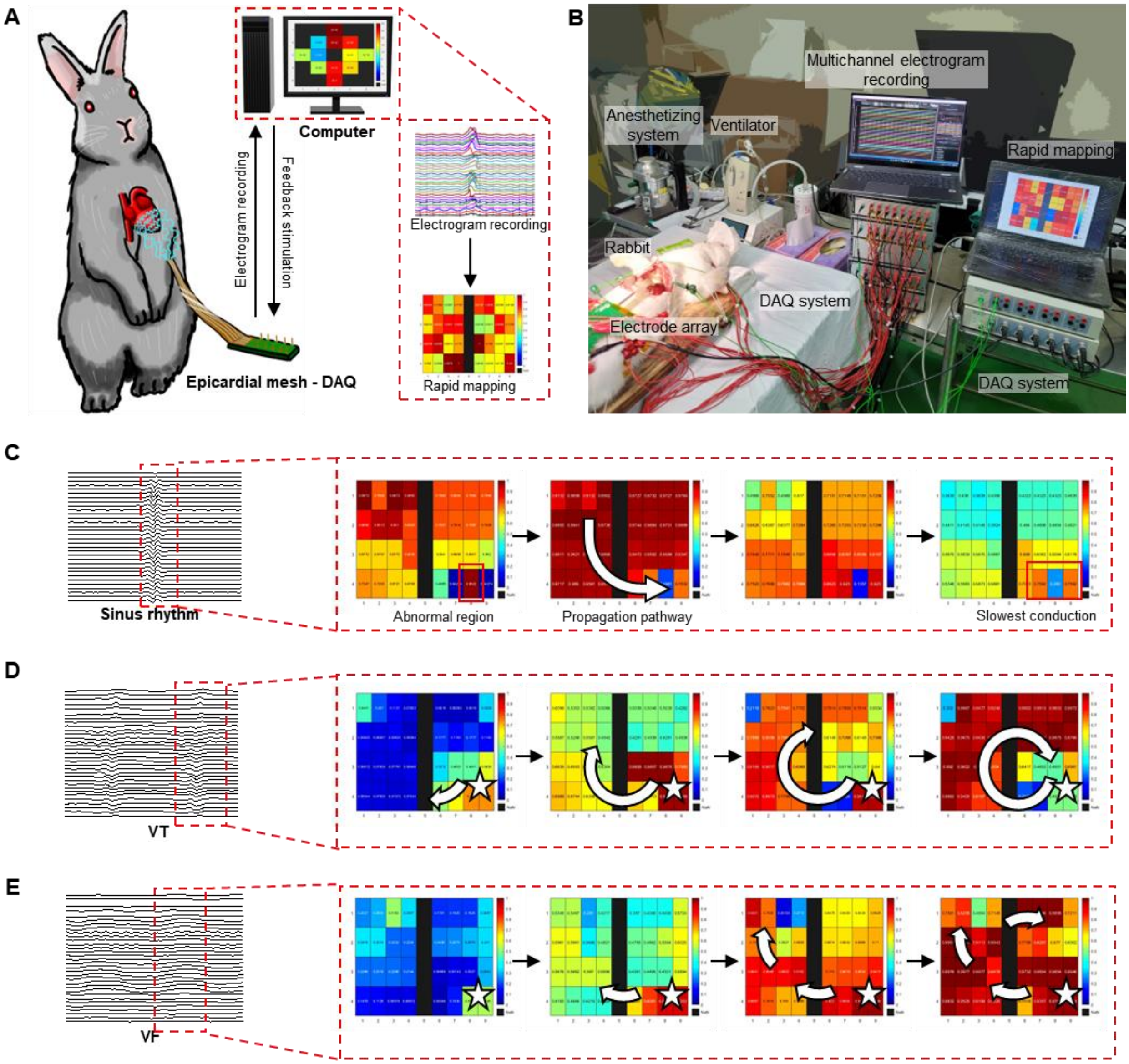

Fig. S4. Programmed rapid mapping using the epicardial 32-channel electrode array. (A) Schematic illustration describing the rapid mapping process during the experiment. **(B)** Optical image of the experimental setting. **(C)** Representative rapid mapping result of the voltage amplitude during sinus rhythm. Abnormally slow conduction was observed in the bottom right region (LV apex). **(D)** Representative rapid mapping result of the voltage amplitude during VT. The re-entrant VT was initiated from the bottom right region (LV apex) and propagated to the left bottom region (RV apex). Then the VT was propagated to the top left (RV base) and top right (LV base) region. **(E)** Representative rapid mapping result of the voltage amplitude during VF. The activation started from the bottom right (LV apex) region and propagated to the neighboring tissue. However, the propagation was fragmented compared to the VT propagation map.

(LV, left ventricle; VT, ventricular tachycardia; RV, right ventricle; VF, ventricular fibrillation)

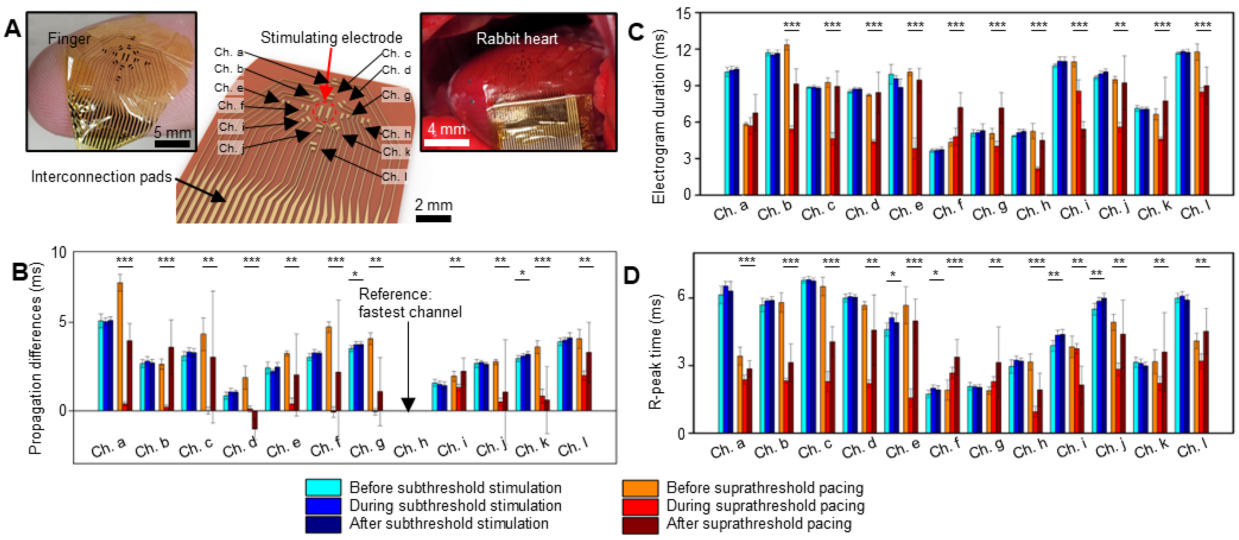

Fig. S5. Characterization of the subthreshold stimulation versus the suprathreshold pacing. (A) Schematic illustration describing the 13-channel electrode array arranged in a concentric shape to study the characteristics of the subthreshold stimulation and suprathreshold pacing. The stimulation electrode is located at the center with 12 electrode channels surrounding the stimulation electrode. **(B-D)** Characteristics of the subthreshold stimulations (blue bars) and suprathreshold pacings (red bars). Propagation delay **(B)**, electrogram durations **(C)**, and R-peak time **(D)** are presented. R-peak time implies the time for the peak from the activation onset. n=8 per channel. $^{*}P$ <0.05, $^{**}P$ <0.01, $^{***}P$ <0.001, comparisons otherwise were not significant.

(ch, channel)

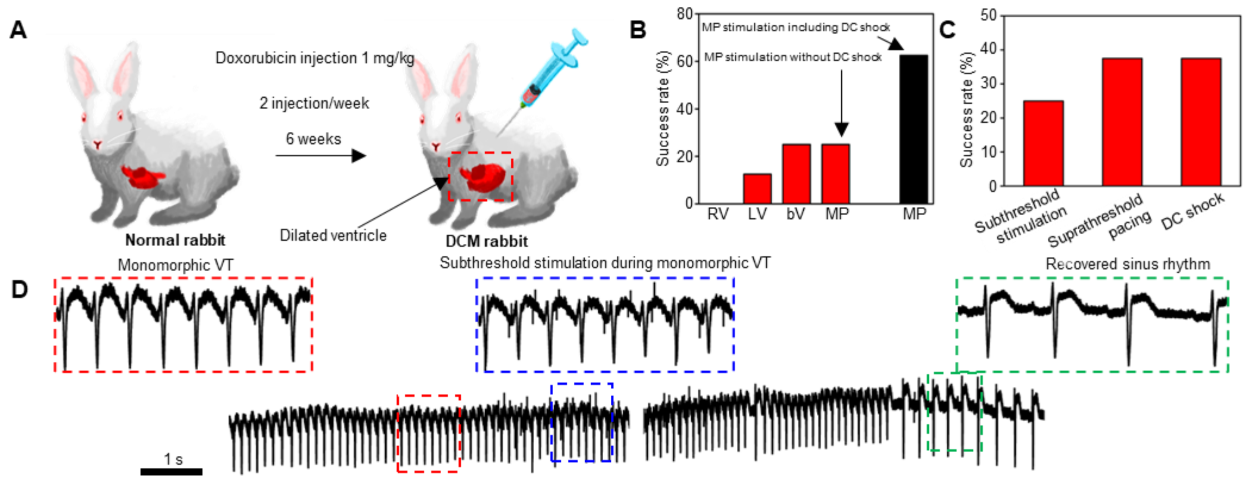

Fig. S6. Ventricular tachyarrhythmia termination protocol performed on the rabbit DCM model. (A) Schematic illustration describing the experimental protocol. **(B)** VTA termination success rate of DCM model by region of stimulation. **(C)** VTA termination success rate of DCM model by type of stimulation/pacing. **(D)** Representative surface ECG of the rabbit during the VT termination protocol using subthreshold stimulation.

(DCM, dilated cardiomyopathy; RV, right ventricle; LV, left ventricle; bV, biventricle; MP, multipoint; DC, direct current; VT, ventricular tachycardia; VTA, ventricular tachyarrhythmia; ECG, electrocardiogram)

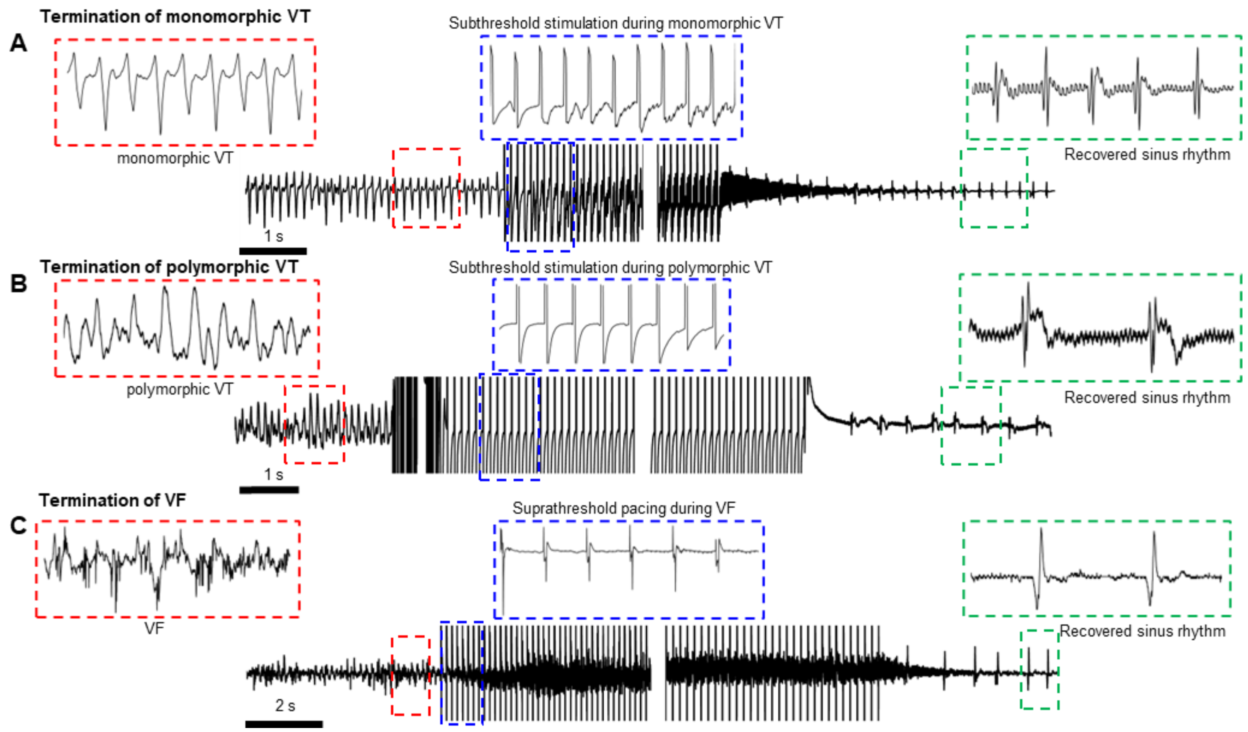

Fig. S7. Termination of various ventricular tachyarrhythmias with the multi-channel electrode array. (A) Termination of monomorphic VT with subthreshold stimulation. Electrogram under VT (red dotted box), stimulation (blue dotted box), and recovered sinus rhythm (green dotted box). **(B)** Termination of polymorphic VT with subthreshold stimulation. **(C)** Termination of VF with suprathreshold pacing.

(VT, ventricular tachycardia; VF, ventricular fibrillation)

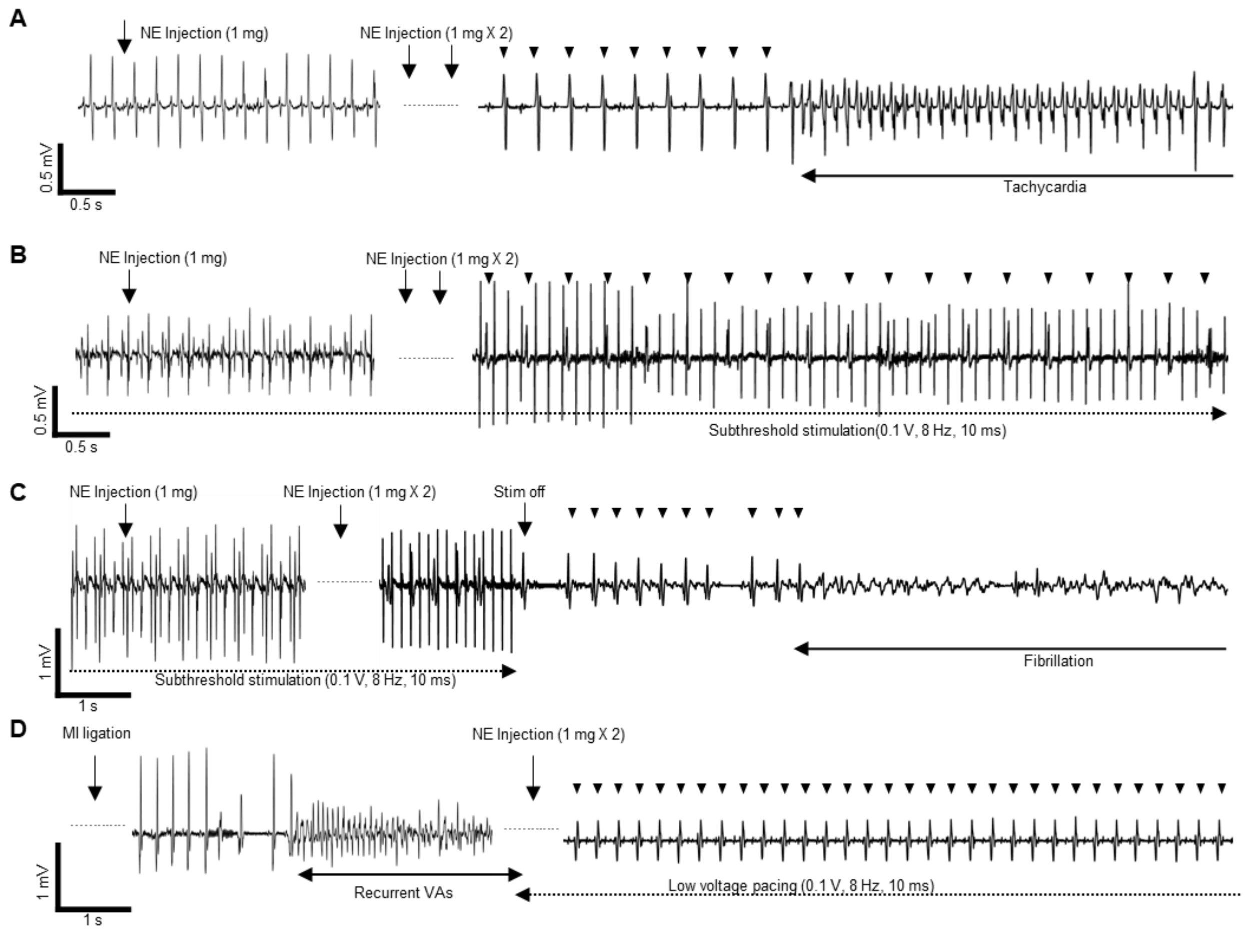

Fig. S8. Prophylactic subthreshold stimulation for ventricular tachyarrhythmia prevention on rat myocardial infarction model. (A) Surface ECG of the MI rat in control group. After NE injection, spontaneous sinus rhythm (black downward triangle) disappeared after the onset of VTA. **(B)** Surface ECG of the MI rat in the prevention group with subthreshold stimulation. Spontaneous sinus rhythms (black downward triangle) was maintained after high dose of NE injection. **(C)** Surface ECG of the MI rat in the prevention group with subthreshold stimulation. VTA event occurred after offset of the subthreshold stimulation (“Stim off”), after 10 beats of sinus rhythm (black downward triangle). **(D)** Surface ECG of the MI rat in the control group. Continuous recurrence of VTA was terminated with subthreshold stimulation and sinus rhythm (black downward triangle) was restored.

(ECG, electrocardiogram; MI, myocardial infarction; NE, norepinephrine; VTA, ventricular tachyarrhythmias; VA, ventricular tachyarrhythmia)

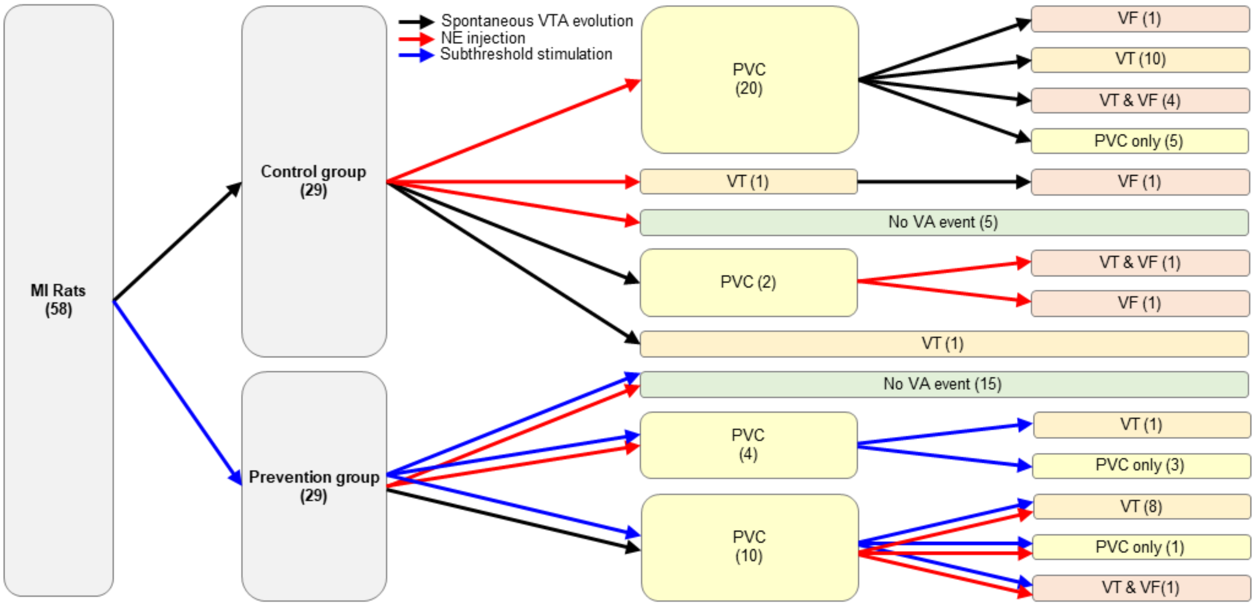

Fig. S9. Incidence of ventricular tachyarrhythmia in the prevention protocols of rat myocardial infarction model. A chart summarizing the detailed sequence of the various VTA events, including the number of each event that occurred during the application of the prevention protocol based on the subthreshold stimulation.

(MI, myocardial infarction; VTA, ventricular tachyarrhythmias; NE, norepinephrine; PVC, premature ventricular contraction; VF, ventricular fibrillation; VT, ventricular tachycardia)

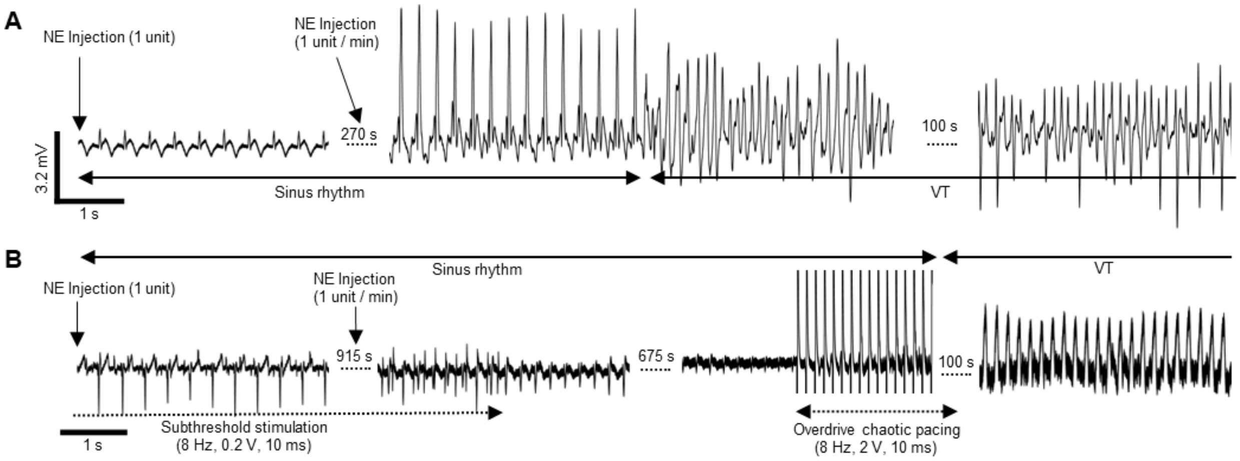

Fig. S10. Prophylactic subthreshold stimulation for ventricular tachyarrhythmia prevention on rabbit myocardial infarction model. (A) Surface ECG of the MI rabbit in the control group. After NE injection, spontaneous sinus rhythm disappeared after the onset of VT. **(B)** Surface ECG of the MI rabbit in the prevention group with subthreshold stimulation. Spontaneous sinus rhythms were maintained even after high dose of NE injection when under subthreshold stimulation. However, with subthreshold stimulation cessation followed by overdrive suprathreshold pacing, VT event occurred.

(ECG, electrocardiogram; MI, myocardial infarction; NE, norepinephrine; VT, ventricular tachycardia)

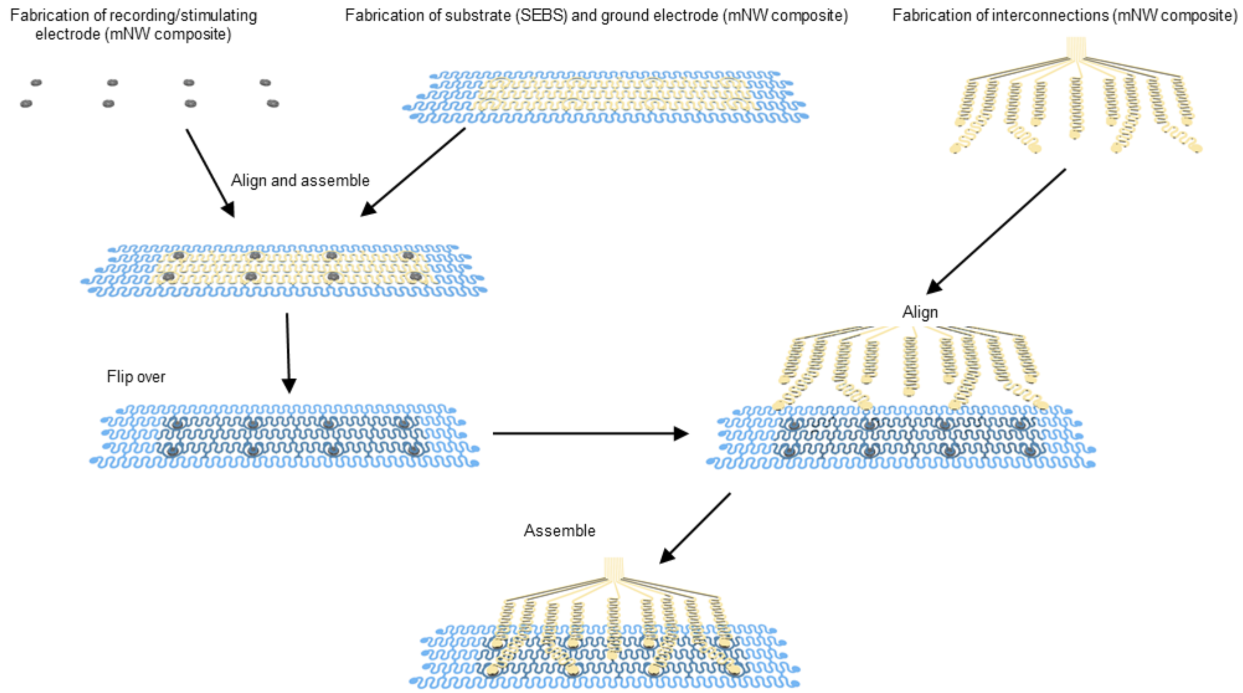

Fig. S11. Integration process of the stretchable epicardial multi-channel electrode array. The recording/stimulating electrode, ground electrode, and substrate layer are independently fabricated and then integrated following the sequence described in this illustration.
(mNW, metal nanowire; SEBS, styrene-ethylene-butylene-styrene)

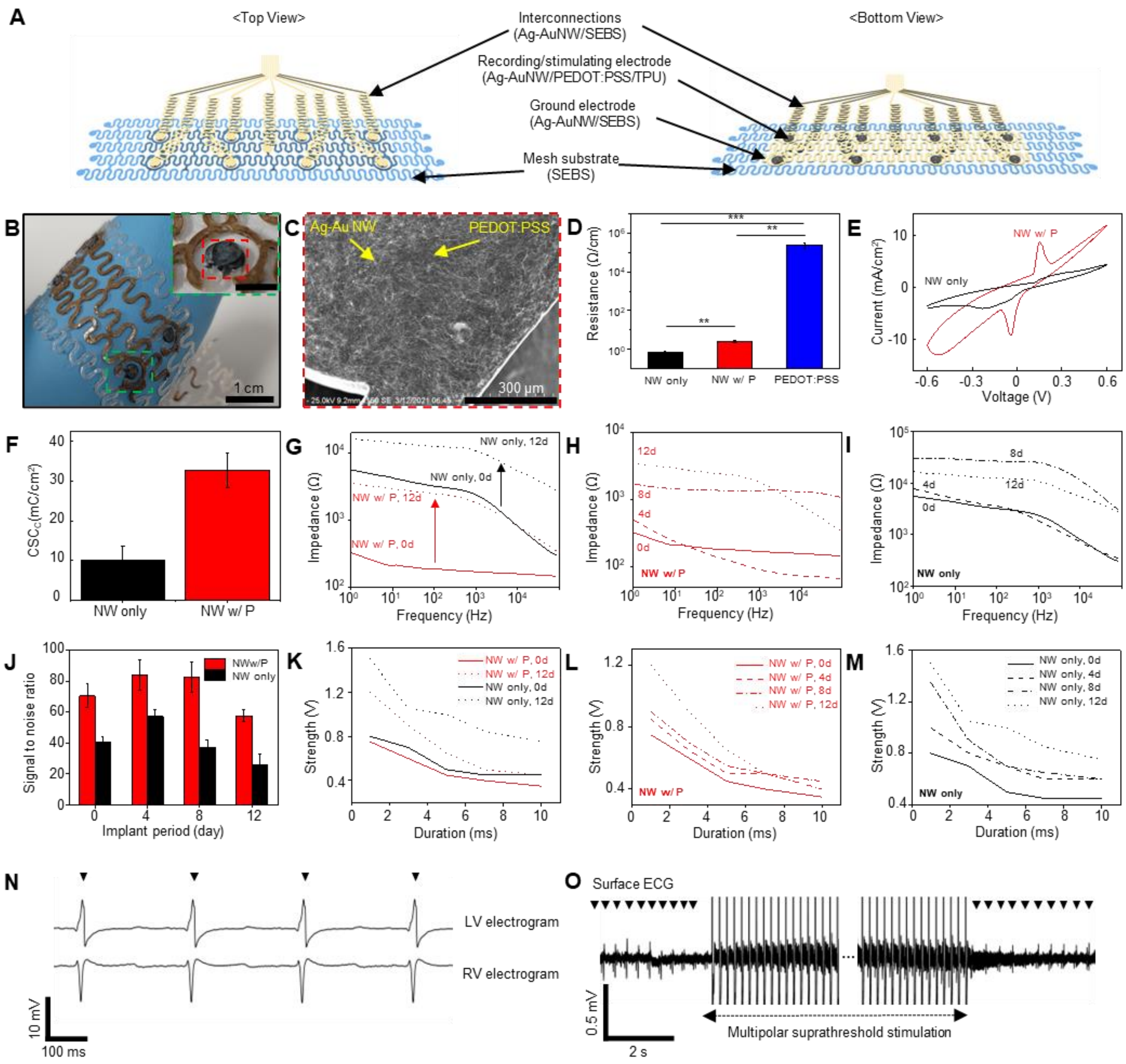

Fig. S12. Electrode array based on the Ag-Au NW/PEDOT:PSS/TPU and Ag-Au NW/SEBS nanocomposites. (A) Design of the stretchable epicardial electrode array using the Ag-Au NW/PEDOT:PSS/TPU nanocomposite (for recording/stimulating electrode) and the Ag-Au NW/SEBS nanocomposite (for ground electrode and interconnection). **(B)** Optical camera image of the electrode array mounted on the thumb. The inset shows a magnified view of the concentric pair that consists of the central recording/stimulating electrode and the circumferential surrounding ground electrode (white scale bar, 2 mm in the inset). **(C)** SEM image of the Ag-Au NW/PEDOT:PSS/TPU nanocomposite. **(D)** Conductivity of Ag-Au NWs (NW only), PEDOT:PSS, and Au NW/PEDOT:PSS/TPU nanocomposites (NW w/P). $^{**}P<0.01$, $^{***}P<0.001$ for pairwise comparison. **(E)** Current-voltage curve of the nanocomposite with PEDOT:PSS (NW w/ P) and without PEDOT:PSS (NW only). **(F)** Charge storage capacity (CSC) comparison between nanocomposite with (red) or without (black) PEDOT:PSS. (**G**) Time-dependent changes in impedance of the nanocomposites with (red) or without (black) PEDOT:PSS. The impedance

12 days after implantation was shown in dotted line. (**H** and **I**) Time-dependent impedance changes of the nanocomposites with (**H**) and without (**I**) PEDOT:PSS, recorded for 12 days, every 4 days. **(J)** Time-dependent changes in signal-to-noise ratio of the nanocomposites with (red bar) or without (black bar) PEDOT:PSS. **(K)** Time-dependent changes in threshold voltage of the nanocomposites with (red) or without (black) PEDOT:PSS. The threshold voltage 12 days after implantation was shown in dotted line. (**L** and **M**) Time-dependent threshold voltage changes of the nanocomposites with (**L**) and without (**M**) PEDOT:PSS, measured for 12 days, every 4 days. **(N)** Intracardiac electrogram recorded using the Ag-Au NW/PEDOT:PSS/TPU electrode on the rabbit heart. Downward triangles indicate sinus peaks. **(O)** Surface electrocardiogram recorded from a rabbit using the Ag-Au NW/PEDOT:PSS/TPU electrode during electrical stimulations. Downward triangles indicate sinus peaks.

(Ag-Au NW, silver-gold core-shell nanowire; ECG, electrocardiogram SEBS, styrene-ethylene-butylene-styrene; PEDOT, poly(3,4-ethylenedioxythiophene):poly(styrene sulfonate; PSS, polystyrene sulfonate; TPU, thermoplastic polyurethane; SEM, scanning electron microscope; LV, left ventricle; RV, right ventricle; NW only, Ag-Au NW only; NW w/ P, Ag-Au NW/PEDOT:PSS/TPU; CSC, charge storage capacity)

3. Supplementary Movies

Movie. S1. Rapid mapping result of a sinus rhythm displayed in a movie. The voltage amplitude of the ventricle was mapped from the signals acquired by the epicardial 32-channel electrode array. Note the spread of the ventricular activation from the right to left ventricle, with abnormally slow conduction in the bottom right region (LV apex), corresponding to the region of MI.

Movie. S2. Rapid mapping result of a re-entrant ventricular tachycardia displayed in a movie. The voltage amplitude of the ventricle was mapped from the signals acquired by the epicardial 32-channel electrode array. Note the VT initiating from the bottom right region (LV apex) and propagating to the left bottom region (RV apex). Then, the VT propagates to the top left (RV base) and top right (LV base) region.

Movie. S3. Rapid mapping result of a ventricular fibrillation displayed in a movie. The voltage amplitude of the ventricle was mapped from the signals acquired by the epicardial 32-channel electrode array. Note the activation of VF starting from the bottom right (LV apex) region and propagated to the neighboring tissue. However, the propagation is much more fragmented compared to the VT propagation map.

REFERENCES AND NOTES

1. E. D. Paratz, L. Rowsell, D. Zentner, S. Parsons, N. Morgan, T. Thompson, P. James, A. Pflaumer, C. Semsarian, K. Smith, D. Stub, A. La Gerche; Australian UCDP Registry, Cardiac arrest and sudden cardiac death registries: A systematic review of global coverage. *Open Heart* **7**, e001195 (2020).

2. J. W. Waks, A. E. Buxton, Risk stratification for sudden cardiac death after myocardial infarction. *Annu. Rev. Med.* **69**, 147–164 (2018).

3. K. Soejima, W. G. Stevenson, Ventricular tachycardia associated with myocardial infarct Scar. *Circulation* **106**, 176–179 (2002).

4. P. S. Chan, R. A. Hayward, Mortality reduction by implantable cardioverter-defibrillators in high-risk patients with heart failure, ischemic heart disease, and new-onset ventricular arrhythmia: An effectiveness study. *J. Am. Coll. Cardiol.* **45**, 1474–1481 (2005).

5. F. Z. Khan, M. S. Virdee, S. P. Fynn, D. P. Dutka, Left ventricular lead placement in cardiac resynchronization therapy: Where and how? *Europace* **11**, 554–561 (2009).

6. M. S. Wathen, P. J. DeGroot, M. O. Sweeney, A. J. Stark, M. F. Otterness, W. O. Adkisson, R. C. Canby, K. Khalighi, C. Machado, D. S. Rubenstein, K. J. Volosin; for the PainFREE Rx II Investigators Prospective randomized multicenter trial of empirical antitachycardia pacing versus shocks for spontaneous rapid ventricular tachycardia in patients with implantable cardioverter-defibrillators. *Circulation* **110**, 2591–2596 (2004).

7. D. Duncker, C. Veltmann, Optimizing antitachycardia pacing: Back to the roots. *Circ. Arrhythm. Electrophysiol.* **10**, e005696 (2017).

8. M. Shenasa, R. Cardinal, T. Kus, P. Savard, M. Fromer, P. Page, Termination of sustained ventricular tachycardia by ultrarapid subthreshold stimulation in humans. *Circulation* **78**, 1135–1143 (1988).

9. G. Salama, A. Kanai, I. R. Efimov, Subthreshold stimulation of Purkinje fibers interrupts ventricular tachycardia in intact hearts: Experimental study with voltage-sensitive dyes and imaging techniques. *Circ. Res.* **74**, 604–619 (1994).

10. M. Shenasa, M. Fromer, M. Borggrefe, G. Breithardt, Subthreshold electrical stimulation for termination and prevention of reentrant tachycardias. *J. Electrocardiol.* **24**, 25–31 (1992).

11. M. Weiss, S. Evans, A. Grunwald, R. Palazzo, U. Neveling, S. Blumberg, M. Bodenheimer, 991-73 ultrarapid subthreshold stimulation delivered via epicardial patches can terminate reentrant ventricular tachycardia in a canine model. *J. Am. Coll. Cardiol.* **25**, 315A (1995).

12. F. S. Ng, O. Toman, J. Petru, P. Peichl, R. A. Winkle, V. Y. Reddy, P. Neuzil, R. H. Mead, N. A. Qureshi, Z. I. Whinnett, D. W. Bourn, M. B. Shelton, J. Kautzner, A. D. Sharma, M. Hocini, M. Haïssaguerre, N. S. Peters, I. R. Efimov, Novel low-voltage multipulse therapy to terminate atrial fibrillation. *JACC Clin. Electrophysiol.* **7**, 988–999 (2021).

13. X. Lin, Y. Liu, A. Bai, H. Cai, Y. Bai, W. Jiang, H. Yang, X. Wang, L. Yang, N. Sun, H. Gao, A viscoelastic adhesive epicardial patch for treating myocardial infarction. *Nat. Biomed. Eng.* **3**, 632–643 (2019).

14. J. Park, S. S. H. Choi, A. H. Janardhan, S.-Y. Y. Lee, S. Raut, J. Soares, K. Shin, S. Yang, C. Lee, K.-W. W. Kang, H. R. Cho, S. J. Kim, P. Seo, W. Hyun, S. Jung, H.-J. J. Lee, N. Lee, S. S. H. Choi, M. Sacks, N. Lu, M. E. Josephson, T. Hyeon, D.-H. Kim, H. J. Hwang, Electromechanical cardioplasty using a wrapped elasto-conductive epicardial mesh. *Sci. Transl. Med.* **8**, 344ra86 (2016).

15. S. Choi, S. I. Han, D. Jung, H. J. Hwang, C. Lim, S. Bae, O. K. Park, C. M. Tschabrunn, M. Lee, S. Y. Bae, J. W. Yu, J. H. Ryu, S.-W. Lee, K. Park, P. M. Kang, W. B. Lee, R. Nezafat, T. Hyeon, D.-H. Kim, Highly conductive, stretchable and biocompatible Ag–Au core–sheath nanowire composite for wearable and implantable bioelectronics. *Nat. Nanotechnol.* **13**, 1048–1056 (2018).

16. S.-H. Sunwoo, S. I. Han, H. Kang, Y. S. Cho, D. Jung, C. Lim, C. Lim, M. jin Cha, S.-P. Lee, T. Hyeon, D.-H. Kim, Stretchable low-impedance nanocomposite comprised of Ag–Au core–shell nanowires and Pt black for epicardial recording and stimulation. *Adv. Mater. Technol.* **5**, 1900768 (2020).

17. A. J. Graham, M. Orini, P. D. Lambiase, Limitations and challenges in mapping ventricular tachycardia: New technologies and future directions. *Arrhythm. Electrophysiol. Rev.* **6**, 118–124 (2017).

18. T. Irie, R. Yu, J. S. Bradfield, M. Vaseghi, E. F. Buch, O. Ajijola, C. Macias, O. Fujimura, R. Mandapati, N. G. Boyle, K. Shivkumar, R. Tung, Relationship between sinus rhythm late activation zones and critical sites for scar-related ventricular tachycardia. *Circ. Arrhythm. Electrophysiol.* **8**, 390–399 (2015).

19. M. T. Roe, E. M. Ohman, A. C. P. Maas, R. H. Christenson, K. W. Mahaffey, C. B. Granger, R. A. Harrington, R. M. Califf, M. W. Krucoff, Shifting the open-artery hypothesis downstream: The quest for optimal reperfusion. *J. Am. Coll. Cardiol.* **37**, 9–18 (2001).

20. W. R. Hathaway, E. D. Peterson, G. S. Wagner, C. B. Granger, K. M. Zabel, K. S. Pieper, K. A. Clark, L. H. Woodlief, R. M. Califf, Prognostic significance of the initial electrocardiogram in patients with acute myocardial infarction. *JAMA* **279**, 387–391 (1998).

21. M. Balli, M. Çetin, H. Taşolar, K. Tekin, Ç. E. Çağliyan, S. Türkmen, O. K. Uysal, M. Çayli, Increased ventricular pacing threshold levels in patients with high serum uric acid levels. *J. Cardiol.* **64**, 207–210 (2014).

22. Y. Tomita, S. Yamada, T. Kaneshiro, N. Hijioka, T. Shimizu, Y. Takeishi, A sudden rise in pacing threshold of left ventricular lead associated with myocardial ischemia. *J. Arrhythm.* **37**, 693–695 (2021).

23. T. Yamada, G. N. Kay, Optimal ablation strategies for different types of ventricular tachycardias. *Nat. Rev. Cardiol.* **9**, 512–525 (2012).

24. O. Hauswirth, D. Noble, R. W. Tsien, Adrenaline: Mechanism of action on the pacemaker potential in cardiac Purkinje fibers. *Science* **162**, 916–917 (1968).

25. M. Haghjoo, M. Hajahmadi, A. F. Fazelifar, M. A. Sadr-Ameli, Efficacy and safety of different antitachycardia pacing sites in the termination of ventricular tachycardia in patients with biventricular implantable cardioverter-defibrillator. *Europace* **13**, 509–513 (2011).

26. M. Fromer, M. Shenasa, Ultrarapid subthreshold stimulation for termination of atrioventricular node reentrant tachycardia. *J. Am. Coll. Cardiol.* **20**, 879–883 (1992).

27. Y. G. Kim, Y. Y. Choi, K.-D. Han, K. J. Min, H. Y. Choi, J. Shim, J.-i. Choi, Y.-H. Kim, Premature ventricular contraction increases the risk of heart failure and ventricular tachyarrhythmia. *Sci. Rep.* **11**, 12698 (2021).

28. C. Theis, H. Mollnau, S. Sonnenschein, T. Konrad, E. Himmrich, K. Bock, E. Schulz, D. Kämpfner, S. Gerhardt, B. Quesada Ocete, T. Münzel, T. Rostock, Reduction of ICD shock burden by eliminating back-up pacing induced ventricular tachyarrhythmias. *J. Cardiovasc. Electrophysiol.* **25**, 889–895 (2014).

29. M. J. Pagnani, P. M. Pellicci, E. A. Salvati, Effect of aspirin on heterotopic ossification after total hip arthroplasty in men who have osteoarthrosis. *J. Bone Joint Surg. Am.* **73**, 924–929 (1991).

30. B. L. Wilkoff, L. Fauchier, M. K. Stiles, C. A. Morillo, S. M. Al-Khatib, J. Almendral, L. Aguinaga, R. D. Berger, A. Cuesta, J. P. Daubert, S. Dubner, K. A. Ellenbogen, N. A. Mark Estes, G. Fenelon, F. C. Garcia, M. Gasparini, D. E. Haines, J. S. Healey, J. L. Hurtwitz, R. Keegan, C. Kolb, K. H. Kuck, G. Marinskis, M. Martinelli, M. McGuire, L. G. Molina, K. Okumura, A. Proclemer, A. M. Russo, J. P. Singh, C. D. Swerdlow, W. S. Teo, W. Uribe, S. Viskin, C. C. Wang, S. Zhang; Document Reviewers, G. Boriani, M. Brignole, A. Cheng, T. C. Crawford, L. D. Biase, K. Donahue, A. E. Epstein, M. E. Field, B. Gorenek, J.-L. Huang, J. H. Indik, C. W. Israel, M. L. Jessup, C. Leclercq, R. J. Macfadyen, C. Madias, M. F. Marquez, B. Olshansky, K. K. Patton, M. M. Refaat, C. M. Tracy, G. A. Upadhyay, D. Vanegas, P. J. Wang,

2015 HRS/EHRA/APHRS/SOLAECE expert consensus statement on optimal implantable cardioverter-defibrillator programming and testing. *EP Europace* **18**, 159–183 (2016).

31. S.-P. Lee, H.-J. Im, S. Kang, S.-J. Chung, Y. S. Cho, H. Kang, H. S. Park, D.-W. Hwang, J.-B. Park, J.-C. Paeng, G.-J. Cheon, Y.-S. Lee, J. M. Jeong, Y.-J. Kim, Noninvasive imaging of myocardial inflammation in myocarditis using ^{68}Ga-tagged mannosylated human serum albumin positron emission tomography. *Theranostics* **7**, 413–424 (2017).

32. J.-B. Park, M. Suh, J. Y. Park, J. K. Park, Y. -I Kim, H. Kim, Y. S. Cho, H. Kang, K. Kim, J. H. Choi, J. W. Nam, H.-K. Kim, Y.-S. Lee, J. M. Jeong, Y.-J. Kim, J. C. Paeng, S.-P. Lee, Assessment of inflammation in pulmonary artery hypertension by ^{68}Ga-mannosylated human serum albumin. *Am. J. Respir. Crit. Care Med.* **201**, 95–106 (2020).

33. C. J. W. Borleffs, R. J. van Bommel, S. G. Molhoek, J. G. de Leeuw, M. J. Schalij, L. Van Erven, Requirement for coronary sinus lead interventions and effectiveness of endovascular replacement during long-term follow-up after implantation of a resynchronization device. *Europace* **11**, 607–611 (2009).

34. C. D. Swerdlow, K. A. Ellenbogen, Implantable cardioverter-defibrillator leads. *Circulation* **128**, 2062–2071 (2013).

35. M. Kotsakou, I. Kioumis, G. Lazaridis, G. Pitsiou, S. Lampaki, A. Papaiwannou, A. Karavergou, K. Tsakiridis, N. Katsikogiannis, I. Karapantzos, C. Karapantzou, S. Baka, I. Mpoukovinas, V. Karavasilis, A. Rapti, G. Trakada, A. Zissimopoulos, K. Zarogoulidis, P. Zarogoulidis, Pacemaker insertion. *Ann. Transl. Med.* **3**, 42 (2015).

36. D. Duncker, T. König, S. Hohmann, J. Bauersachs, C. Veltmann, Avoiding untimely implantable cardioverter/defibrillator implantation by intensified heart failure therapy optimization supported by the wearable cardioverter/defibrillator-the PROLONG study. *J. Am. Heart Assoc.* **6**, e004512 (2017).

37. Z. F. Issa, S. S. Rumman, J. C. Mullin, Inadvertent transarterial insertion of atrial and ventricular defibrillator leads. *J. Interv. Card. Electrophysiol.* **24**, 63–66 (2009).

38. B. J. Pang, S. S. Barold, H. G. Mond, Injury to the coronary arteries and related structures by implantation of cardiac implantable electronic devices. *Europace* **17**, 524–529 (2015).

39. M. O. Sweeney, L. Sherfesee, P. J. DeGroot, M. S. Wathen, B. L. Wilkoff, Differences in effects of electrical therapy type for ventricular arrhythmias on mortality in implantable cardioverter-defibrillator patients. *Heart Rhythm* **7**, 353–360 (2010).

40. Y. Fang, K. Gu, B. Yang, W. Ju, H. Chen, M. Li, H. Liu, J. Wang, G. Yang, M. Chen, What factors lead to the acceleration of ventricular tachycardia during antitachycardia pacing?—Results from over 1000 episodes. *J. Arrhythm* **34**, 36–45 (2018).

41. P. Neuzil, M. Taborsky, Z. Rezek, R. Vopalka, L. Sediva, P. Niederle, V. Reddy, Pacemaker and ICD lead extraction with electrosurgical dissection sheaths and standard transvenous extraction systems: Results of a randomized trial. *Europace* **9**, 98–104 (2007).

42. A. Sugrue, C. V. DeSimone, C. J. Lenz, D. L. Packer, S. J. Asirvatham, Mobile thrombus on cardiac implantable electronic device leads of patients undergoing cardiac ablation: Incidence, management, and outcomes. *J. Interv. Card. Electrophysiol.* **46**, 115–120 (2016).

43. N. A. McKeag, D. J. McEneaney, Thrombus formation on a defibrillator lead with conductor externalization. *Indian Pacing Electrophysiol. J.* **15**, 143–144 (2015).

44. M. Banaszewski, J. Stępińska, Right heart perforation by pacemaker leads. *Arch. Med. Sci.* **8**, 11–13 (2012).

45. A. P. Vanezis, R. Prasad, R. Andrews, Pacemaker leads and cardiac perforation. *JRSM Open* **8**, 205427041668143 (2017).

46. J. C. Hsu, P. D. Varosy, H. Bao, T. A. Dewland, J. P. Curtis, G. M. Marcus, Cardiac perforation from implantable cardioverter-defibrillator lead placement: Insights from the National Cardiovascular Data Registry. *Circ. Cardiovasc. Qual. Outcomes* **6**, 582–590 (2013).

47. R. R. M. Vogels, A. Lambertz, P. Schuster, S. Jockenhoevel, N. D. Bouvy, C. Disselhorst-Klug, U. P. Neumann, U. Klinge, C. D. Klink, Biocompatibility and biomechanical analysis of elastic

TPU threads as new suture material. *J. Biomed. Mater. Res. B Appl. Biomater.* **105**, 99–106 (2017).

48. V. Jašo, M. V. Rodić, Z. S. Petrović, Biocompatible fibers from thermoplastic polyurethane reinforced with polylactic acid microfibers. *Eur. Polym. J.* **63**, 20–28 (2015).

49. C.-H. Chen, A. Kine, R. D. Nelson, J. C. LaRue, Impedance spectroscopy study of conducting polymer blends of PEDOT:PSS and PVA. *Synth. Met.* **206**, 106–114 (2015).

50. D. A. Koutsouras, P. Gkoupidenis, C. Stolz, V. Subramanian, G. G. Malliaras, D. C. Martin, Impedance spectroscopy of spin-cast and electrochemically deposited PEDOT:PSS films on microfabricated electrodes with various areas. *ChemElectroChem* **4**, 2321–2327 (2017).

51. I. Nuramdhani, A. T. Gokceoren, S. A. Odhiambo, G. D. De Mey, C. Hertleer, L. Van Langenhove, Electrochemical impedance analysis of a PEDOT: PSS-based textile energy storage device. *Materials (Basel)* **11**, 48 (2018).

52. H.-Y. Mi, X. Jing, B. N. Napiwocki, B. S. Hagerty, G. Chen, L.-S. Turng, Biocompatible, degradable thermoplastic polyurethane based on polycaprolactone-block-polytetrahydrofuran-block-polycaprolactone copolymers for soft tissue engineering. *J. Mater. Chem. B Mater. Biol. Med.* **5**, 4137–4151 (2017).
